# Development and validation of a prognostic model for stage IV breast cancer based on primary tumor resection with machine learning methods: retrospective cohort study

**DOI:** 10.3389/fendo.2026.1871537

**Published:** 2026-07-13

**Authors:** Yaoling Wang, Jingyu Hou, Xinhai Chen, Hongyi Zhu, Yuan Yao, Wenjing Zhao, Shuangwei Mo, Zhenchong Xiong, Anli Yang, Wei Liu, Yuanhui Lai, Weikai Xiao

**Affiliations:** 1 Department of Thyroid and Breast Surgery, The Affiliated Guangdong Second Provincial General Hospital of Jinan University, Guangzhou, Guangdong, China; 2 Department of Breast, Guangdong Provincial People’s Hospital and Guangdong Academy of Medical Sciences, Southern Medical University, Guangzhou, China; 3 School of Medicine, South China University of Technology, Guangzhou, China; 4 Guangdong Lung Cancer Institute, Guangdong Provincial People’s Hospital, Guangdong Academy of Medical Sciences, Southern Medical University, Guangzhou, Guangdong, China; 5 Department of Medical Oncology, State Key Laboratory of Oncology in South China, Sun Yat-Sen University Cancer Center, Guangzhou, China; 6 Department of Breast Oncology, Sun Yat-sen University Cancer Center, State Key Laboratory of Oncology in South China, Guangdong Provincial Clinical Research Center for Cancer, Guangzhou, China; 7 Department of Breast Surgery, Guangzhou Red Cross Hospital of Jinan University, Guangzhou, Guangdong, China

**Keywords:** machine learning, primary tumor resection (PTR), prognostic web application, stage IV breast cancer, survival prediction

## Abstract

**Background:**

Primary Tumor Resection (PTR) remains controversial among women with stage IV breast cancer.

**Objective:**

Using a machine learning (ML) approach, this study investigates how PTR is associated with survival outcomes in stage IV breast cancer. We aim to develop a model capable of identifying patient characteristics linked to better prognosis following PTR, ultimately providing a data-informed tool to support prognostic assessment and clinical evaluation.

**Methods:**

A propensity-score matched analysis of stage IV breast cancer patients in the SEER registry (2000-2020) was conducted, using Cox regression and Kaplan-Meier methods to estimate overall survival (OS) and cancer-specific survival (CSS). Among five ML models, an internally and externally validated ML model was crafted for predicting survival outcomes, with a user-friendly web platform based on the shiny platform for clinical use.

**Results:**

In a cohort of 10,194 stage IV breast cancer patients, with 5,732 matched subjects, Cox regression analysis showed PTR’s positive associations with OS (HR, 0.61; 95% CI, 0.57 to 0.66) and CSS (HR, 0.64; 95% CI, 0.59 to 0.67). Subgroup analysis indicated better survival for patients with tumors ≤5 cm, N2 status (OS, HR, 0.52; 95% CI, 0.44 - 0.63;CSS,HR, 0.52; 95% CI, 0.43 - 0.63), and HER2 overexpression (OS, HR, 0.56; 95% CI, 0.52 - 0.61;CSS,HR, 0.56; 95% CI, 0.46 - 0.67), especially those without systemic treatment. The best outcomes were seen with trimodality therapy combining PTR, chemotherapy, and radiotherapy (OS, HR, 0.40; 95% CI, 0.37 - 0.44; CSS, HR, 0.03; 95% CI, 0.01 - 0.10). The Support Vector Machine (SVM) model (6-month: AUC = 0.935; 1-year: AUC = 0.945; 2-year: AUC = 0.941; 3-year: AUC = 0.921) was identified as the most precise tool for survival prediction, demonstrating high accuracy and consistency across external datasets. Furthermore, a user-friendly web application was created to make the prognostic model more accessible.

**Conclusions:**

Patients with tumors ≤5 cm, N2 status, or HER2 overexpression were associated with better survival after PTR. Our ML model, which is based on eight clinical indicators, predicts survival and assists in identifying surgical candidates for stage IV cancer.

## Introduction

Breast cancer remains the preeminent malignancy among women in the United States, projected to account for nearly a third of all female cancer cases in 2025, with anticipated new diagnoses and mortalities reaching 319,750 and 42,000, respectively (1). By January 1, 2025, the metastatic breast cancer (MBC) landscape is expected to encompass 169,347 cases (2). Since the 1990s, despite the proliferation of mammography and enhanced treatment modalities, a subset of 6% to 10% of breast cancer patients still present with metastatic disease (3), and the incidence rate of distant metastasis has increased by 0.7% every year (1, 4). Compared to early-stage breast cancer (EBC), MBC, characterized by its incurability and attenuated survival rates, is underscoring an urgent need for refined treatment strategies that may include systemic therapies and localized regional treatments tailored to individual patient disease status (5, 6).

The efficacy of primary tumor resection (PTR) as a therapeutic intervention is not universally applicable to the metastatic breast cancer patient population; however, it holds potential advantages for a select cohort. Delineating the specific subset of patients who stand to benefit from PTR represents an area of research ripe for exploration, with implications for personalized treatment strategies and enhanced clinical outcomes.

Divergent findings within the academic sphere have yet to coalesce around a unified stance on the utility of PTR for stage IV breast cancer. While some studies (7–10), especially a meta-analysis (11) of 67,272 patients and the 1:1 multicenter, randomized clinical trial MF07-01 (12) insinuate a survival advantage conferred by PTR, others, including the prospective phase III ABCSG-28 trial (13), refute its impact on overall survival (OS). The ambiguity extends to the delineation of patient groups that might achieve enhanced survival outcomes from surgical intervention, particularly in the context of bone metastasis (14–16) and hormone receptor (HR) or HER2 positive status (7, 17).

As diagnostics and therapeutics for breast cancer advance, the necessity for deeper investigative forays into the surgical impact on stage IV breast cancer patient stratifications becomes imperative. With the advancement of pharmaceuticals and their contribution to the prognosis of breast cancer, it is imperative to re-evaluate existing data to align with the progress of contemporary medicine.

In this study, our objective was to develop and validate a system to assist clinicians in identifying stage IV breast cancer patients who are associated with favorable survival outcomes after PTR. Utilizing the Surveillance, Epidemiology, and End Results (SEER) database, we compiled a real-world dataset of patients with stage IV breast cancer, mitigating selection bias through 1:1 propensity score matching (PSM). Employing univariable and multivariable Cox regression analyses along with subgroup analysis, we identified characteristics associated with favorable survival outcomes among stage IV breast cancer patients who underwent PTR. After meticulously examining correlations with these characteristics on survival prognostication, we leveraged various machine learning algorithms to identify the optimal predictive model, which was subsequently validated for its predictive performance within a cohort from the Sun Yat-sen University Cancer Center (SYSUCC). To facilitate clinical application, this system was further implemented as a user-friendly web-based application for identifying patients who may be associated with favorable survival outcomes following PTR.

## Methods

### Patient selection

**Figure 1:**
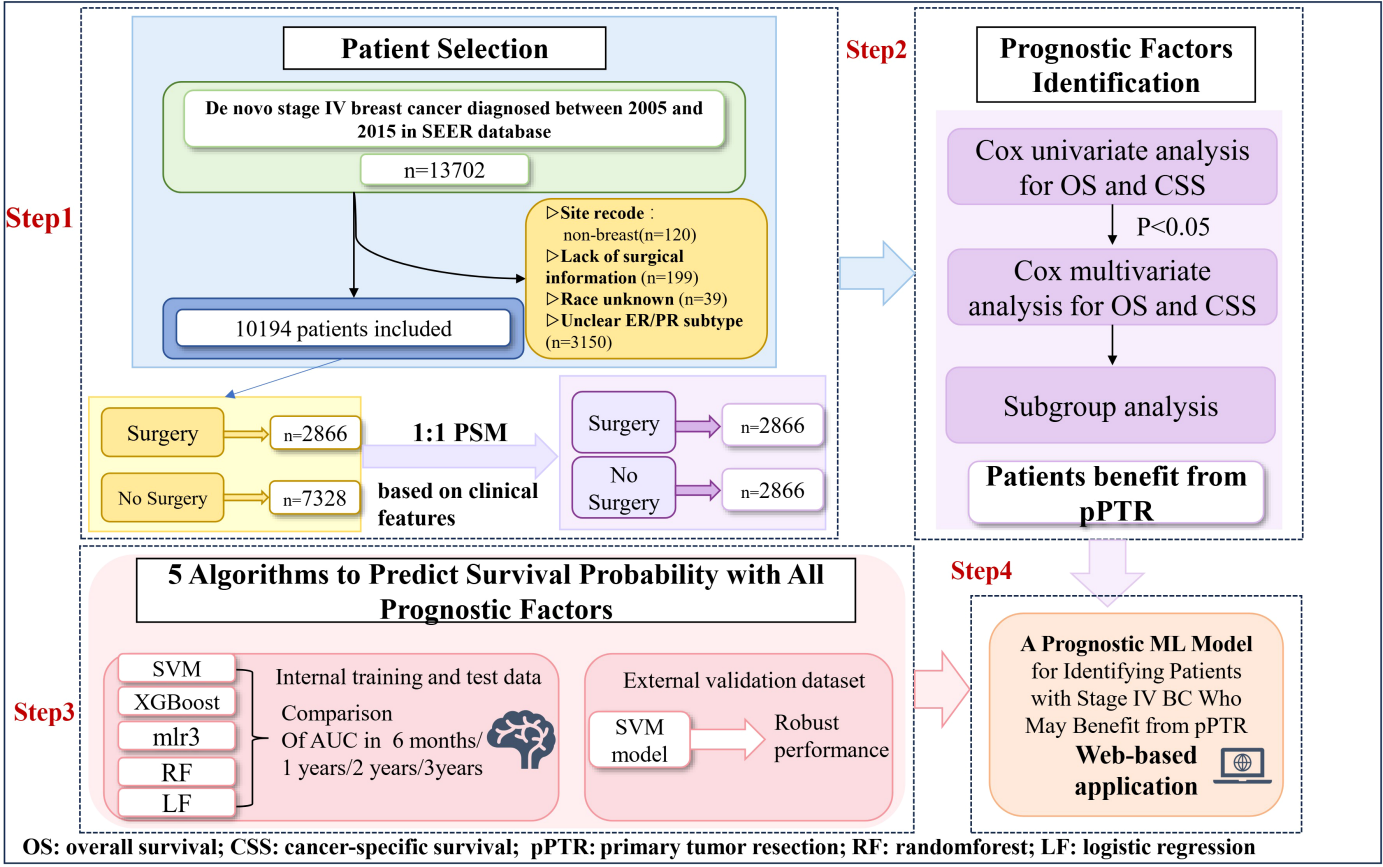
Study workflow for developing a machine learning model to identify patients with *de novo* stage IV breast cancer who may benefit from primary tumor resection. This retrospective cohort study analyzed data from 10,194 patients diagnosed between 2005 and 2015 in the SEER database. After propensity score matching, balanced surgical and non-surgical cohorts (each n=2,866) were established. The workflow included: (1) prognostic factor identification via Cox regression; (2) evaluation of five machine learning algorithms for survival prediction; (3) selection of the optimal model (SVM); and (4) development of a final prognostic model and web application.

This study is based on the Surveillance, Epidemiology, and End Results (SEER) database, published April, 2023 [SEER Research Data, 17 Registries, Nov 2022 Sub (2000–2020 release)] was analyzed as a data source. We extracted clinicopathological and survival information using SEER*Stat software (version 8.4.3). Given the profound epidemiological, biological, and therapeutic distinctions between male and female breast cancer, this study focused exclusively on female patients to ensure clinical homogeneity and statistical validity. Data about women with BC were collected from this database. Patients were included according to the following criteria: (1) diagnosis of stage IV breast cancer; (2) female sex; and (3) diagnosis between 2005 and 2015. This study period was selected to ensure sufficient follow-up for survival analyses while maintaining an adequate sample size. This exclusion is standard practice to ensure data integrity and model interpretability, as an ‘unknown’ category does not represent a biologically or clinically meaningful group and could introduce misclassification bias. In addition, we excluded patients who met the following criteria: (1) non-breast primary sites were excluded to maintain disease-specific analytical focus; (2) patients missing primary surgery data were excluded as this represents a fundamental treatment variable;(3) patients with unknown race were excluded to prevent misclassification bias in demographic analyses; (4) patients with unknown ER/PR status were excluded as these biomarkers are essential for contemporary breast cancer classification and treatment stratification (**Figure 1**). A sensitivity analysis was conducted to assess potential selection bias from excluding patients with unknown race. Comparisons of key baseline characteristics between the included and excluded cohorts revealed no significant differences (**Supplementary Table 1**).

**Figure 2:**
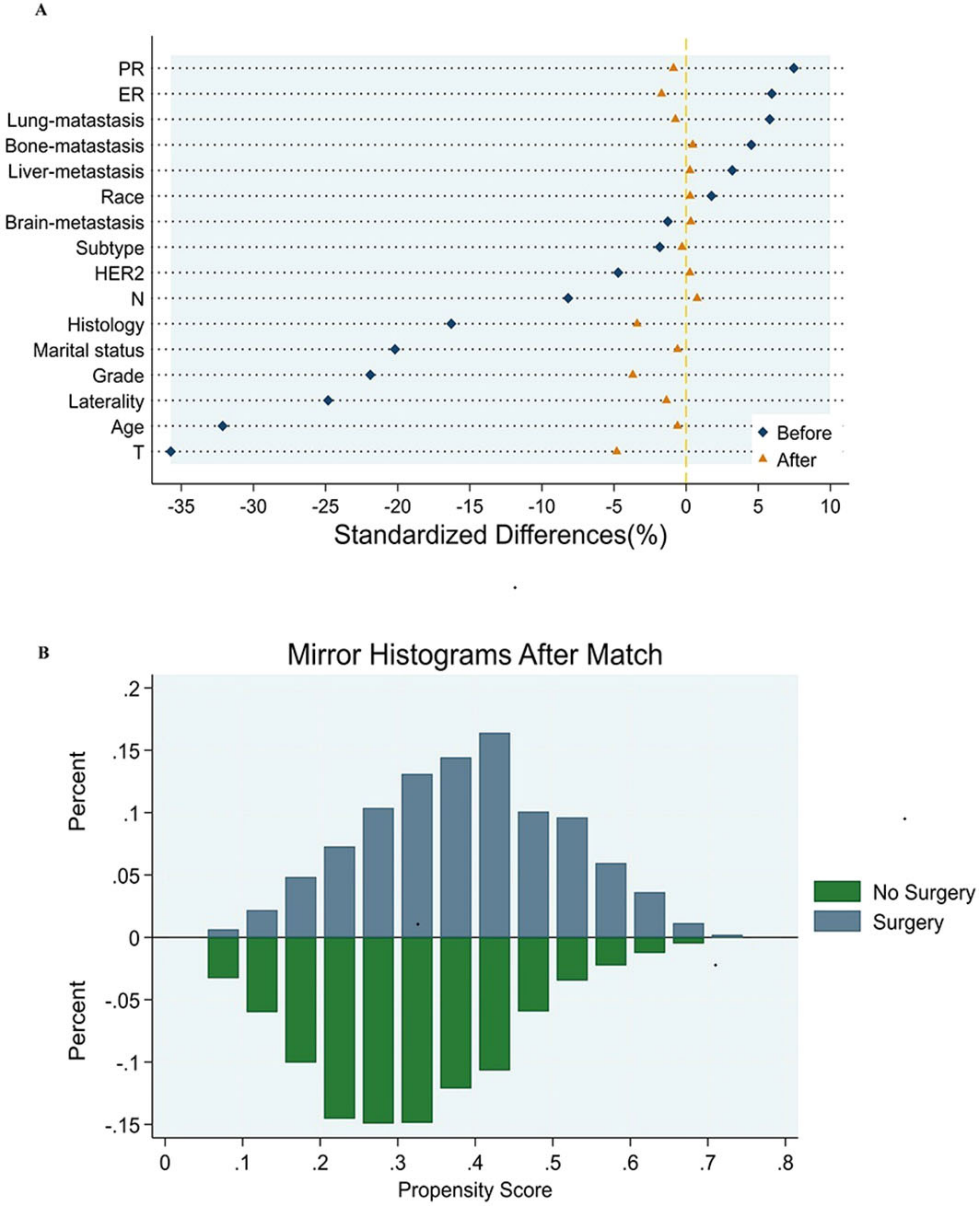
Propensity-matched analysis. **(A)** Standardized differences before and after the match. **(B)** Mirror histogram.

To approximate randomization and mitigate selection bias, we fitted a logistic regression model that included all relevant clinical confounders to estimate propensity scores. Using these scores, we performed 1:1 propensity score matching between the surgery and non-surgery cohorts, without replacement and with each patient included only once. We chose variables through a combination of clinical relevance and statistical significance in existing studies. Variables used for matching included: age, race, TNM category of tumor, subtype of breast cancer, histological grade and histological type, marital status, presence or absence of bone, brain, liver and lung metastasis, and use of radiotherapy or chemotherapy. Standardized mean differences with mirror histograms after matching are illustrated in **Figure 2**.

In this study, the external dataset was approved by the Ethics Committee of the Cancer Centre of Sun Yat-sen University, with the approval number SL-G2023-300-01.

### Model construction

To predict patient survival status at specific time points, we constructed five distinct binary classification models. Each model was trained to predict survival at a predefined landmark time: 6 months, 1 year, 2 years, and 3 years after surgery. Model performance was evaluated based on the Area Under the ROC Curve (AUC) and Accuracy. Furthermore, to assess the accuracy of the predicted probabilities, we performed calibration analysis by plotting calibration curves and calculating the Brier score. To quantify the relative informative improvement of our machine learning model over a baseline naive null model, the scaled Brier score was further computed. The 95% confidence intervals (CIs) for both the overall and scaled Brier scores were estimated using a bootstrap resampling procedure with 2,000 replicates. Additionally, Decision Curve Analysis (DCA) was performed to evaluate the clinical utility and net benefit of the developed model against default clinical strategies. The model demonstrating optimal performance on the validation set was selected as the final model.

This study was reported in accordance with the TRIPOD+AI (Transparent Reporting of a multivariable prediction model for Individual Prognosis Or Diagnosis using Artificial Intelligence) recommendations (18).

#### SVM model

The Support Vector Machine (SVM) is a powerful supervised learning algorithm used for both classification and regression (19, 20). It operates by finding the optimal hyperplane that best separates the data into different classes, maximizing the margin between the closest points of the classes, which are known as support vectors. There are several advantages of SVM:(1) maximization of margin: the principle of maximizing the margin between different classes leads to better generalization on unseen data, making SVMs less prone to overfitting;(2) robustness to noise: SVMs with soft margins are tolerant to noise and outliers in the data, which is a practical advantage in real-world datasets. (3) effectiveness in small sample sizes.

#### XGBoost model

The Extreme Gradient Boosting (XGBoost) model is an optimized distributed gradient boosting library, is known for its efficiency and performance in handling large datasets with complex relationships (21). It is adept at managing the survival prediction by effectively dealing with censored data, a common challenge in time-to-event analysis. XGBoost can be tuned to optimize its predictive accuracy by adjusting parameters such as the learning rate, the number of trees, and the depth of the trees.

#### Random forest model

The Random Forest model extends the decision tree approach by creating an ensemble of trees to predict survival outcomes. They are particularly effective in capturing complex interactions between variables and can be used for dynamic predictions that incorporate longitudinal information over time (19). It is lauded for its ability to handle high-dimensional data and its robustness to overfitting. In survival analysis, Random Forest can capture complex interactions between features and is effective in predicting survival outcomes.

#### Logistic regression model

Logistic Regression is a statistical method for binary classification tasks, where it estimates the probability that a given sample belongs to a particular class. It is particularly useful in survival analysis for predicting the likelihood of an event occurring within a specified time frame. The logistic function ensures that the model’s predictions stay within the bounds of probability, making it a principled approach for binary outcomes (21, 22).

#### mlr3 model

The mlr3 package in R is a comprehensive framework designed for machine learning tasks, including regression, classification, and survival analysis. It provides a unified interface for training, testing, and evaluating a wide range of machine learning algorithms. The flexibility and extensibility of mlr3 make it an advantageous tool for survival prediction, as it supports complex experimental designs and automates many aspects of the machine learning workflow (21, 23).

#### Hyperparameter tuning

For all models requiring hyperparameter optimization (including SVM, RF, XGBoost, and LR), we employed Grid Search combined with 5-fold Cross-Validation to determine the optimal hyperparameter combination. Model performance was assessed based on Accuracy.

**Figure 3:**
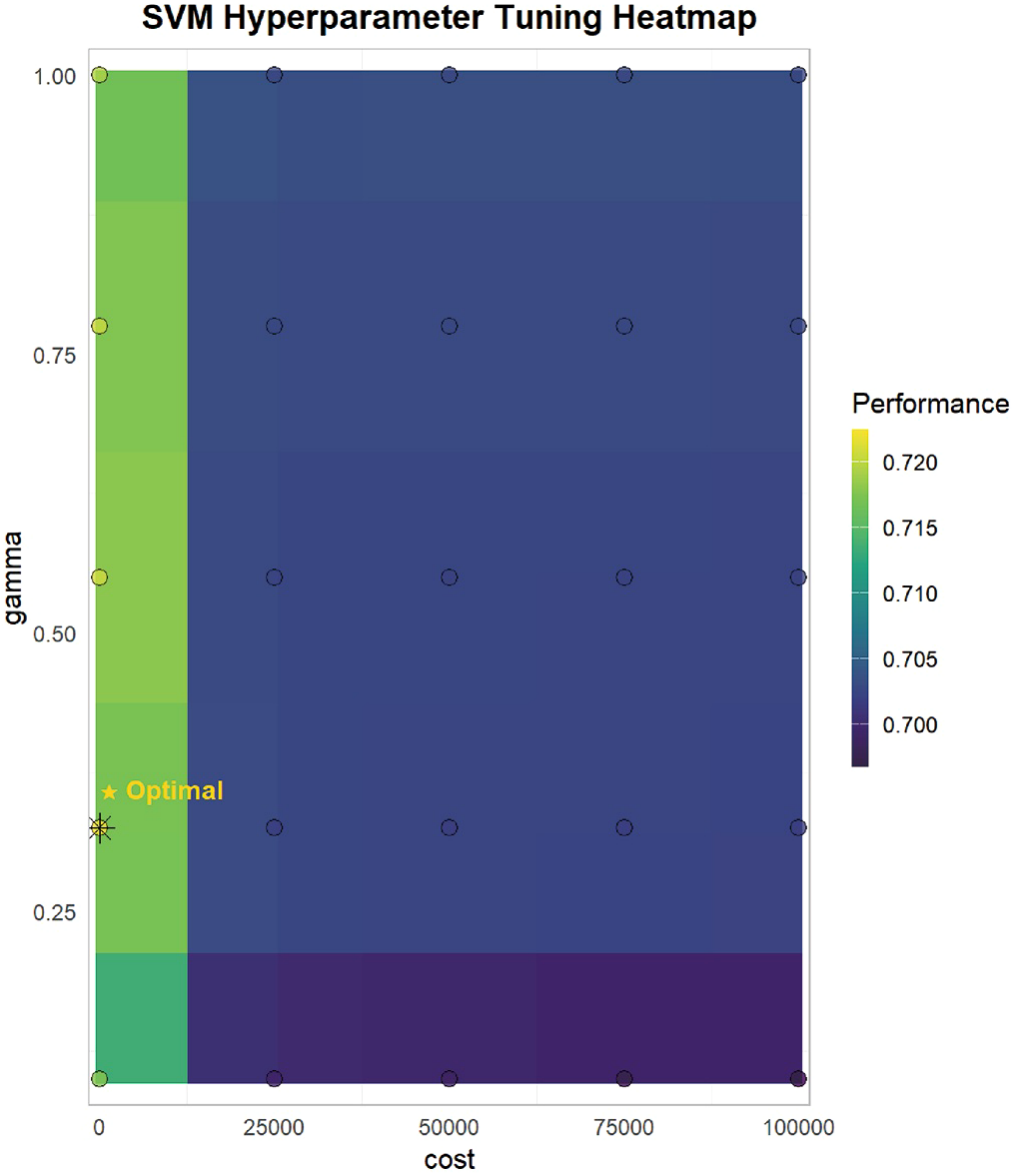
Visualization of hyperparameter optimization trajectories and final parameter selection for the SVM model. Heatmap depicting mean Accuracy across validation folds for different cost and gamma combinations during Grid Search. The optimal parameter combination (cost=0.1, gamma=0.325) is marked with a yellow asterisk.

The hyperparameter tuning process was conducted with the training set, utilizing 5-fold cross-validation to evaluate performance across parameter combinations and mitigate overfitting risks. **Figure 3** visually presents the hyperparameter tuning result for the SVM model.

#### Feature selection and internal validation

The selection of features for the final machine learning model was guided by statistical analysis and clinical rationale to ensure robustness and interpretability. We screened clinically relevant variables through univariable and multivariable Cox regression analyses. Tumor size, despite being available in eight categories, was excluded because it is inherently encoded within the T-category variable, which was included in the model. Radiotherapy and grade were omitted due to profound sample imbalance, which risked introducing substantial bias. Marital status was considered a socio-demographic variable rather than a direct biological prognostic factor. The final clinical covariate set—age, cancer subtype, T and N category, ER, PR, HER2 status, chemotherapy receipt, and PTR—was fed into ML models trained to predict OS at 6 months and at 1, 2, and 3 years.

For this study, we deployed five distinct machine learning algorithms: SVM, XGBoost, RF, LR, and the mlr3 package. Patients were systematically allocated into training and testing datasets in a 7:3 ratio. For each prediction horizon, patients censored before the corresponding time point were excluded because their survival status at the target time could not be definitively determined. Models were subsequently developed using only patients with known outcomes at each landmark time point. The efficacy of our models was evaluated using Receiver Operating Characteristic (ROC) analysis, with performance quantified by the Area Under the Curve (AUC) and accuracy metrics, ensuring a rigorous assessment of their predictive capabilities.

#### External validation

In order to further validate the prognostic model, we used clinicopathological and survival information on 67 metastatic breast cancer patients between January 2006 and April 2013 at Sun Yet-sen University Cancer Center. Patients were eligible for inclusion if they had (1) pathological diagnosis of breast cancer; (2) metastatic disease at the time of diagnosis of breast cancer or developed metastasis within 3 months of diagnosis; and (3) life expectancy > 6 months. Patients were excluded if they (1) had incomplete follow-up data or (2) had history of previous cancer or synchronous malignant tumors. Ethical approval for the study was obtained from the institutional review board. The 95% CIs of the AUCs for the external validation cohort were calculated using the bootstrap method with 2,000 resamples.

### SHAP (SHapley Additive exPlanations) analysis

To interpret the predictive model and quantify the marginal contribution of each feature to individual predictions, we employed SHAP analysis. The analysis was conducted in R version 4.4.1 using the fastshap package. SHAP values were calculated for each observation in the test data using the explain() function. The global importance of a feature was determined by averaging the absolute SHAP values across all evaluated instances. The results are visualized using a SHAP summary plot, which displays feature importance (mean |SHAP value|) alongside the distribution of each feature’s impact (SHAP value) on the model output (24, 25).

### Development of the web application

Our web application was meticulously crafted using the R, with a particular emphasis on the Shiny package—a powerful tool widely recognized for its ability to transform R scripts into engaging, interactive web applications. The application is designed to compare the predicted survival rates of patients who underwent surgery with those who did not. By doing so, it provides insights into the potential benefits of surgery in terms of survival outcomes. This interface allows users to easily input patient data, enabling sophisticated predictions of survival probabilities for stage IV breast cancer patients.

### Statistical analysis

StataMP 18, SPSS 26.0 and R-4.4.1 were used for all analyses in this study. All statistical tests were bilateral, and *P* <.05 was considered statistically significant. We performed 1:1 PSM without replacement by the nearest-neighbor algorithm with a caliper of 0.1 times the standard deviation of the propensity score. Standardized differences were used to examine the balance across baseline covariates before and after matching. A standardized difference of less than 0.10 (10%) was considered indicative of good balance (26). Then, the differences of variables between the surgery and non-surgery groups (before and after PSM) were evaluated by the χ² test. We determined OS and CSS as two primary endpoints in this research. OS was measured as the time from BC diagnosis to the date of death due to any cause (including BC) or the date of last follow-up. CSS was measured as the time from the date of BC diagnosis to the date of BC death or the date of last follow-up. Subsequently, the Kaplan–Meier (K-M) method with the log-rank test was generated to observe the differences in OS and CSS between the surgery and non-surgery groups. Furthermore, we conducted univariable, multivariable Cox regression analyses, along with the assessment of multicollinearity among the variables using the variance inflation factor (VIF). Subgroup analyses were conducted to evaluate the independent protective effect of primary tumor resection. These analyses were also employed to pinpoint the independent clinicopathological factors associated with CSS. While the SEER database records broad treatment categories without detailed regimen information, we performed a stratification analysis to assess the impact of changing practices (2005-2015). The study is also subject to immortal time bias inherent to observational data, which cannot be fully addressed due to the lack of precise treatment timelines.

## Results

### Baseline characteristics

In this study, 10,194 patients diagnosed with BC between 2005 and 2015 met our selection criteria for further analysis. Of these, 2866 patients received surgical treatment, while 7328 did not receive PTR (**Table 1**). We then performed a PSM analysis and matched 5732 patients on a 1:1 basis, including surgical and non-surgical cohort. 4462 patients were excluded in this process. In the matched cohort, the distribution of baseline features was well-balanced, as depicted in **Figure 2** and detailed in **Table 2**. The standardized differences for all measured covariates were reduced below the 0.10 threshold, demonstrating successful achievement of balance between the surgical and non-surgical groups. It is noted that for some variables (e.g., age, laterality), the p-value from the inter-group comparison remained below 0.05. This is likely attributable to the reduced sample size in the matched cohort affecting the power of the significance test, rather than a meaningful imbalance, as evidenced by the very low corresponding standardized differences (26).

**Table 1:** Baseline characteristic of the unmatched cohort.

Variable	Levels	Non-surgery (N = 7328)	Surgery (N = 2866)	*P*
Age	<45	613 (8.4%)	464 (16.2%)	<.001
	45-65	3436 (46.9%)	1386 (48.4%)	
	>65	3279 (44.7%)	1016 (35.5%)	
Race	White	5593 (76.3%)	2121 (74%)	.04
	Black	1164 (15.9%)	478 (16.7%)	
	Asian	523 (7.1%)	241 (8.4%)	
	American Indian	48 (0.7%)	26 (0.9%)	
Marital status	Married	2944 (40.2%)	1348 (47%)	<.001
	Single	1651 (22.5%)	587 (20.5%)	
	Divorced/Separated/Widowed	2309 (31.5%)	797 (27.8%)	
	Unknown	424 (5.8%)	134 (4.7%)	
Grade	1	300 (4.1%)	155 (5.4%)	<.001
	2	1809 (24.7%)	847 (29.6%)	
	3	2126 (29%)	1438 (50.2%)	
	4	54 (0.7%)	26 (0.9%)	
	Unknown	3039 (41.5%)	400 (14%)	
Laterality	Left	3266 (44.6%)	1446 (50.5%)	<.001
	Right	3086 (42.1%)	1382 (48.2%)	
	Single-sided but unknown	90 (1.2%)	4 (0.1%)	
	Paired sides	886 (12.1%)	34 (1.2%)	
Histology	IDC	4059 (55.4%)	1953 (68.1%)	<.001
	ILC	803 (11%)	280 (9.8%)	
	Mixed	320 (4.4%)	278 (9.7%)	
	others	2146 (29.3%)	355 (12.4%)	
Subtype	HR+/HER2-	2636 (36%)	811 (28.3%)	<.001
	HR+/HER2+	646 (8.8%)	242 (8.4%)	
	HR-/HER2-	526 (7.2%)	231 (8.1%)	
	HR-/HER2+	403 (5.5%)	161 (5.6%)	
	Unknown	3117 (42.5%)	1421 (49.6%)	
T	0	483 (6.6%)	16 (0.6%)	<.001
	1	399 (5.4%)	323 (11.3%)	
	2	755 (10.3%)	676 (23.6%)	
	3	620 (8.5%)	430 (15%)	
	4	2817 (38.4%)	1202 (41.9%)	
	X	2254 (30.8%)	219 (7.6%)	
N	0	1614 (22%)	497 (17.3%)	<.001
	1	2725 (37.2%)	945 (33%)	
	2	540 (7.4%)	499 (17.4%)	
	3	845 (11.5%)	709 (24.7%)	
	X	1604 (21.9%)	216 (7.5%)	
ER	+	5503 (75.1%)	1998 (69.7%)	<.001
	-	1825 (24.9%)	868 (30.3%)	
PR	+	4391 (59.9%)	1565 (54.6%)	<.001
	-	2937 (40.1%)	1301 (45.4%)	
HER2	+	1049 (14.3%)	403 (14.1%)	<.001
	-	3162 (43.1%)	1042 (36.4%)	
	Unknown	3117 (42.5%)	1421 (49.6%)	
Size	Unknown	3378 (46.1%)	388 (13.5%)	<.001
	≤1cm	139 (1.9%)	104 (3.6%)	
	1-2cm	446 (6.1%)	300 (10.5%)	
	2-3cm	459 (6.3%)	372 (13%)	
	3-4cm	429 (5.9%)	279 (9.7%)	
	4-5cm	416 (5.7%)	250 (8.7%)	
	5-8cm	856 (11.7%)	526 (18.4%)	
	>8cm	1205 (16.4%)	647 (22.6%)	
Nodal status	Unknown	1614 (22%)	497 (17.3%)	<.001
	Internal mamary node(s)	35 (0.5%)	18 (0.6%)	
	Micrometastasized axillary lymph node(s)	13 (0.2%)	62 (2.2%)	
	Mobile axillary lymph node(s)	1353 (18.5%)	976 (34.1%)	
	Fixed axillary lymph node(s)	1355 (18.5%)	660 (23%)	
	Infraclavicular node(s)	2958 (40.4%)	653 (22.8%)	
Bone-metastasis	Yes	3152 (43%)	851 (29.7%)	<.001
	No	1362 (18.6%)	632 (22.1%)	
	Unknown	2814 (38.4%)	1383 (48.3%)	
Brain-metastasis	Yes	451 (6.2%)	68 (2.4%)	<.001
	No	3937 (53.7%)	1397 (48.7%)	
	Unknown	2940 (40.1%)	1401 (48.9%)	
Liver-metastasis	Yes	1258 (17.2%)	282 (9.8%)	<.001
	No	3175 (43.3%)	1196 (41.7%)	
	Unknown	2895 (39.5%)	1388 (48.4%)	
Lung-metastasis	Yes	3461 (47.2%)	1172 (40.9%)	<.001
	No	1977 (27%)	841 (29.3%)	
	Unknown	1890 (25.8%)	853 (29.8%)	
Systemic treatment	No systemic or surgical procedure	5442 (74.3%)	348 (12.1%)	<.001
	Introperative therapy	1 (0%)	2 (0.1%)	
	Systemic therapy before surgery	44 (0.6%)	563 (19.6%)	
	Systemic therapy after surgery	821 (11.2%)	995 (34.7%)	
	Surgery both before and after systemic therapy	23 (0.3%)	428 (14.9%)	
	Unknown	997 (13.6%)	530 (18.5%)	
Radiotherapy	No	6978 (95.2%)	1661 (58%)	<.001
	Yes	350 (4.8%)	1205 (42%)	
Chemotherapy	Yes	3406 (46.5%)	1882 (65.7%)	<.001
	No	3922 (53.5%)	984 (34.3%)	

χ2 statistics were used to compare patient characteristics in the unmatched cohort. Two-tailed *P*-values <.05 were assessed as statistically significant.

Data source: Female patients diagnosed with stage IV breast cancer from 2005 to 2015 in the SEER database. ER, estrogen receptor status; PR, progesterone receptor status; HER2, HER-2 receptor status; Grade: pathological stage.

**Table 2:** Baseline characteristic of the matched cohort.

Variable	Levels	Non-surgery (N = 2866)	Surgery (N = 2866)	*P*	*SMD*
Age	<45	381 (13.3%)	464 (16.2%)	.003	0.035
	45-65	1485 (51.8%)	1386 (48.4%)		
	>65	1000 (34.9%)	1016 (35.5%)		
Race	White	2103 (73.4%)	2121 (74%)	.94	0.009
	Black	495 (17.3%)	478 (16.7%)		
	Asian	243 (8.5%)	241 (8.4%)		
	American Indian	25 (0.9%)	26 (0.9%)		
Marital status	Married	1263 (44.1%)	1348 (47%)	.004	0.019
	Single	702 (24.5%)	587 (20.5%)		
	Divorced/Separated/Widowed	771 (26.9%)	797 (27.8%)		
	Unknown	130 (4.5%)	134 (4.7%)		
Grade	1	199 (6.9%)	155 (5.4%)	<.001	0.016
	2	1053 (36.7%)	847 (29.6%)		
	3	1052 (36.7%)	1438 (50.2%)		
	4	16 (0.6%)	26 (0.9%)		
	Unknown	546 (19.1%)	400 (14%)		
Laterality	Left	1625 (56.7%)	1446 (50.5%)	<.001	0.152
	Right	1145 (40%)	1382 (48.2%)		
	Single-sided but unknown	8 (0.3%)	4 (0.1%)		
	Paired sides	88 (3.1%)	34 (1.2%)		
Histology	IDC	2016 (70.3%)	1953 (68.1%)	<.001	0.792
	ILC	274 (9.6%)	280 (9.8%)		
	Mixed	123 (4.3%)	278 (9.7%)		
	others	453 (15.8%)	355 (12.4%)		
Subtype	HR+/HER2-	862 (30.1%)	811 (28.3%)	.34	0.177
	HR+/HER2+	249 (8.7%)	242 (8.4%)		
	HR-/HER2-	203 (7.1%)	231 (8.1%)		
	HR-/HER2+	173 (6%)	161 (5.6%)		
	Unknown	1379 (48.1%)	1421 (49.6%)		
T	0	164 (5.7%)	16 (0.6%)	<.001	0.071
	1	297 (10.4%)	323 (11.3%)		
	2	491 (17.1%)	676 (23.6%)		
	3	310 (10.8%)	430 (15%)		
	4	1088 (38%)	1202 (41.9%)		
	X	516 (18%)	219 (7.6%)		
N	0	669 (23.3%)	497 (17.3%)	<.001	0.001
	1	998 (34.8%)	945 (33%)		
	2	217 (7.6%)	499 (17.4%)		
	3	431 (15%)	709 (24.7%)		
	X	551 (19.2%)	216 (7.5%)		
ER	+	2002 (69.9%)	1998 (69.7%)	.93	0.003
	-	864 (30.1%)	868 (30.3%)		
PR	+	1556 (54.3%)	1565 (54.6%)	.83	0.006
	-	1310 (45.7%)	1301 (45.4%)		
HER2	+	422 (14.7%)	403 (14.1%)	.52	0.030
	-	1065 (37.2%)	1042 (36.4%)		
	Unknown	1379 (48.1%)	1421 (49.6%)		
Size	Unknown	941 (32.8%)	388 (13.5%)	<.001	0.375
	≤1cm	83 (2.9%)	104 (3.6%)		
	1-2cm	286 (10%)	300 (10.5%)		
	2-3cm	236 (8.2%)	372 (13%)		
	3-4cm	235 (8.2%)	279 (9.7%)		
	4-5cm	208 (7.3%)	250 (8.7%)		
	5-8cm	385 (13.4%)	526 (18.4%)		
	>8cm	492 (17.2%)	647 (22.6%)		
Nodal status	Unknown	669 (23.3%)	497 (17.3%)	<.001	0.035
	Internal mamary node(s)	25 (0.9%)	18 (0.6%)		
	Micrometastasized axillary lymph node(s)	7 (0.2%)	62 (2.2%)		
	Mobile axillary lymph node(s)	545 (19%)	976 (34.1%)		
	Fixed axillary lymph node(s)	618 (21.6%)	660 (23%)		
	Infraclavicular node(s)	1002 (35%)	653 (22.8%)		
Bone-metastasis	Yes	933 (32.6%)	851 (29.7%)	.006	0.030
	No	544 (19%)	632 (22.1%)		
	Unknown	1389 (48.5%)	1383 (48.3%)		
Brain-metastasis	Yes	145 (5.1%)	68 (2.4%)	<.001	0.028
	No	1288 (44.9%)	1397 (48.7%)		
	Unknown	1433 (50%)	1401 (48.9%)		
Liver-metastasis	Yes	362 (12.6%)	282 (9.8%)	<.001	0.026
	No	1086 (37.9%)	1196 (41.7%)		
	Unknown	1418 (49.5%)	1388 (48.4%)		
Lung-metastasis	Yes	1264 (44.1%)	1172 (40.9%)	<.001	0.002
	No	653 (22.8%)	841 (29.3%)		
	Unknown	949 (33.1%)	853 (29.8%)		
Systemic treatment	No systemic or surgical procedure	2006 (70%)	348 (12.1%)	<.001	0.989
	Introperative therapy	1 (0%)	2 (0.1%)		
	Systemic therapy before surgery	18 (0.6%)	563 (19.6%)		
	Systemic therapy after surgery	345 (12%)	995 (34.7%)		
	Surgery both before and after systemic therapy	9 (0.3%)	428 (14.9%)		
	Unknown	487 (17%)	530 (18.5%)		
Radiotherapy	No	2712 (94.6%)	1661 (58%)	<.001	0.955
	Yes	154 (5.4%)	1205 (42%)		
Chemotherapy	Yes	1478 (51.6%)	1882 (65.7%)	<.001	0.289
	No	1388 (48.4%)	984 (34.3%)		

χ2 statistics were used to compare patient characteristics in the matched cohort. Two-tailed *P*-values <.05 were assessed as statistically significant. SMD: standardized mean differences.

Data source: Female patients diagnosed with stage IV breast cancer from 2005 to 2015 in the SEER database.ER, estrogen receptor status; PR, progesterone receptor status; HER2, HER-2 receptor status; Grade: pathological stage.

### Correlations of PTR on overall survival with cancer-specific survival

**Figure 4:**
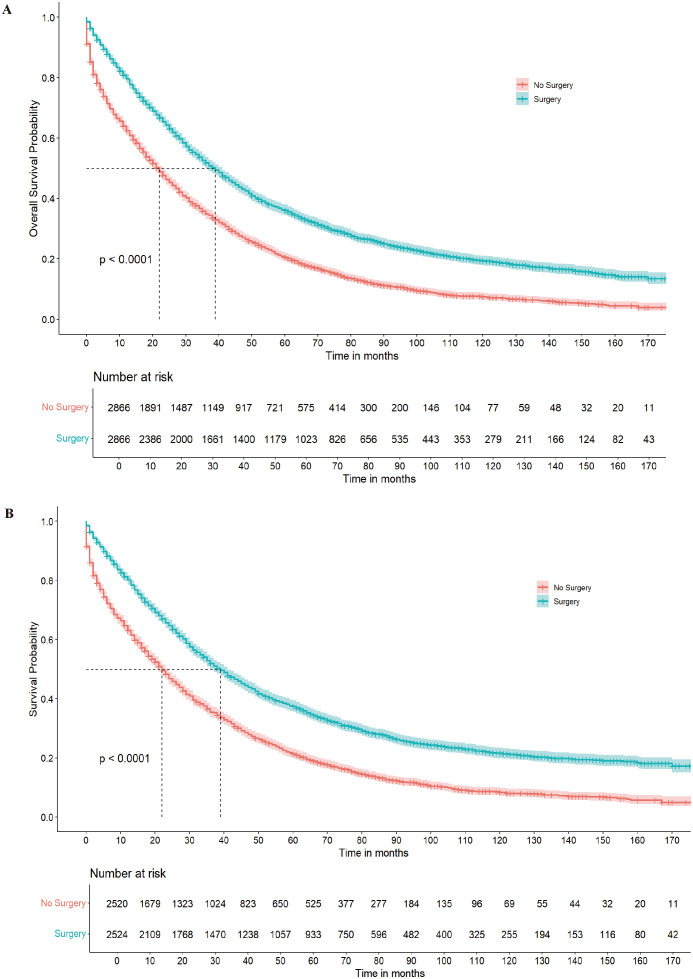
Kaplan-Meier-curves for overall and cancer-specific survival in patients with and without primary cancer resection. **(A)** Overall survival. **(B)** Cancer-specific survival.

The median OS for those who received PTR was 45 months (95% CI, 42 months to 48 months) and 26 months (95% CI, 24 months to 28 months) for those who did not (*χ²* = 294, *P* <.001) (**Figure 4A**). We performed a univariable Cox proportional hazards regression analysis in the matched population, the baseline characteristics including age, race, marital status, laterality, histology, subtype, metastasis in different site(including only bone metastases, only brain metastases, only liver metastases, only lung metastases, oligometastases, multiplemetastases), T category, N category, ER status, PR status, HER2 status, chemotherapy, radiotherapy, PTR, systemic treatment and treatment type. Variables not significantly associated with OS in univariate analysis (including race, metastasis, and histology) were excluded. Furthermore, to mitigate multicollinearity, the overlapping variables “Systemic therapy,” “Treatment type,” and “Nodal metastasis status” were also removed. All other significant variables were entered into the multivariable Cox model. After multivariable risk adjusting in the Cox regression analysis, PTR was a statistically significant protective factor for OS (HR, 0.61; 95% CI, 0.57 to 0.66, *P* <.001) (**Table 3**). Besides, age, tumor grade, marital status, subtype, T category, N category, ER status, PR status, HER2 status, the receipt of chemotherapy and radiotherapy were validated as independent risk or protective factors as well.

**Table 3:** The results of univariable and multivariable Cox regression for clinical characteristics in overall survival. .

Variable	Univariable			Multivariable		
	Crude HR	95%CI	*P*	Adjusted HR	95%CI	*P*
Age			<.001			
<45	[reference]			[reference]		
45-65	1.136	[1.035,1.246]	.01	1.121	[1.021,1.230]	.02
>65	1.317	[1.191,1.456]	<.001	1.295	[1.167,1.437]	<.001
Race			.96			
White	[reference]					
Black	1.324	[1.221,1.435]	<.001			
Asian	0.832	[0.740,0.935]	.002			
American India	0.846	[0.600,1.192]	.34			
Marital status			<.001			
Married	[reference]			[reference]		
Single	1.225	[1.132,1.326]	<.001	1.218	[1.124,1.318]	<.001
Divorced/Separated/Widowed	1.154	[1.069,1.245]	<.001	1.141	[1.057,1.232]	.001
Unknown	1.182	[1.017,1.373]	.03	1.169	[1.006,1.359]	.04
Laterality			.09			
Left	[reference]					
Right	0.945	[0.888,1.006]	.08			
Single-sided but unknown	1.416	[0.761,2.635]	.27			
Paired sides	0.831	[0.663,1.041]	.11			
Histology			.30			
IDC	[reference]					
ILC	1.010	[0.911,1.120]	.85			
Mixed	0.795	[0.703,0.899]	<.001			
Others	1.136	[1.039,1.241]	.005			
Grade			<.001			
1	[reference]			[reference]		
2	1.201	[1.041,1.387]	.01	1.251	[1.083,1.446]	.002
3-4	1.587	[1.379,1.826]	<.001	1.651	[1.426,1.926]	<.001
Unknown	1.313	[1.127,1.530]	<.001	1.382	[1.180,1.620]	<.001
ER			<.001			
ER+	[reference]			[reference]		
ER-	1.393	[1.255,1.546]	<.001	1.391	[1.252,1.545]	<0.001
PR			<.001			
PR+	[reference]			[reference]		
PR-	1.369	[1.258,1.488]	<.001	1.346	[1.237,1.465]	<.001
HER2			<.001			
HER2+	[reference]			[reference]		
HER2-	1.493	[1.344,1.658]	<.001	1.751	[1.568,1.955]	<.001
Unknown	1.651	[1.493,1.827]	<.001	1.749	[1.576,1.940]	<.001
Subtype			<.001			
HR+/HER2-	[reference]			[reference]		
HR+/HER2+	0.726	[0.635,0.829]	<.001	0.721	[0.629,0.828]	<.001
HR-/HER2-	2.303	[2.039,2.602]	<.001	1.245	[1.071,1.446]	.004
HR-/HER2+	0.832	[0.713,0.970]	.02	0.491	[0.411,0.587]	<.001
Unknown	1.304	[1.213,1.402]	<.001	1.054	[0.973,1.141]	.20
T			<.001			
0	[reference]			[reference]		
1	1.451	[1.169,1.801]	.001	1.564	[1.166,2.096]	.003
2	1.606	[1.308,1.972]	<.001	1.935	[1.499,2.498]	<.001
3	1.837	[1.488,2.267]	<.001	1.882	[1.478,2.396]	<.001
4	2.192	[1.799,2.670]	<.001	2.252	[1.809,2.805]	<.001
X	1.791	[1.455,2.204]	<.001	1.790	[1.443,2.221]	<.001
N			<.001			
0	[reference]			[reference]		
1	1.043	[0.953,1.142]	.36	1.036	[0.946,1.134]	.49
2	1.057	[0.941,1.186]	.35	1.036	[0.923,1.164]	.55
3	1.021	[0.921,1.132]	.69	1.009	[0.909,1.119]	.87
X	1.277	[1.143,1.426]	<.001	1.280	[1.146,1.429]	<.001
Nodal Status			<.001			Not included
Unknown	[reference]					
Internal mamary node(s)	1.159	[0.822,1.633]	.40			
Micrometastasized axillary lymph node(s)	0.475	[0.336,0.674]	<.001			
Mobile axillary lymph node(s)	0.909	[0.829,0.997]	.04			
Fixed axillary lymph node(s)	1.112	[1.011,1.224]	.03			
Metastasis			.91			
Only bone metastases	[reference]					
Only brain metastases	2.357	[1.349,4.119]	.003			
Only liver metastases	0.990	[0.654,1.500]	.96			
Only lung metastases	1.042	[0.758,1.431]	.80			
Oligometastases (num<4)	1.153	[0.852,1.561]	.36			
Multplemetastases (num>=4)	1.899	[1.374,2.624]	<.001			
Unknown	1.114	[0.830,1.495]	.47			
Size			.006			
Unknown	[reference]			[reference]		
<1cm	0.732	[0.606,0.884]	.001	1.011	[0.780,1.311]	.93
1-2cm	0.743	[0.660,0.836]	<.001	1.016	[0.835,1.237]	.87
2-3cm	0.694	[0.617,0.781]	<.001	0.854	[0.721,1.012]	.07
3-4cm	0.816	[0.724,0.921]	.001	0.953	[0.808,1.124]	.57
4-5cm	0.872	[0.769,0.989]	.03	1.016	[0.863,1.196]	.85
5-8cm	0.941	[0.853,1.039]	.23	1.076	[0.948,1.222]	.26
>8cm	1.146	[1.047,1.255]	.003	1.186	[1.057,1.330]	.004
Surgery			<.001			
No	[reference]			[reference]		
Yes	0.606	[0.570,0.645]	<.001	0.614	[0.571,0.661]	<.001
Radiotherapy			<.001			
No	[reference]			[reference]		
Yes	0.652	[0.606,0.702]	<.001	0.843	[0.775,0.916]	<.001
Chemotherapy			<.001			
No	[reference]			[reference]		
Yes	0.729	[0.685,0.775]	<.001	0.684	[0.637,0.735]	<.001
Treatment Type			<.001			Not included
No therapy	[reference]					
Surgery only	0.663	[0.595,0.739]	<.001			
Radiation only	0.594	[0.430,0.822]	.002			
Chemotherapy only	0.786	[0.721,0.856]	<.001			
Surgery and chemotherapy	0.522	[0.473,0.576]	<.001			
Surgery and radiation	0.581	[0.502,0.673]	<.001			
Trimodality	0.430	[0.388,0.477]	<.001			
Systemic therapy			<.001			Not included
No systemic therapy	[reference]					
Systemic therapy alone	1.005	[0.324,3.119]	.99			
Surgery after systemic therapy	0.545	[0.488,0.610]	<.001			
Surgery before systemic therapy	0.606	[0.560,0.657]	<.001			
Surgery before and after systemic therapy	0.421	[0.368,0.480]	<.001			
Unknown	0.846	[0.778,0.920]	<.001			

HR, hazard ratio.

Data source: Female patients diagnosed with stage IV breast cancer from 2005 to 2015 in the SEER database. ER+/-, ER-positive/negative status; PR+/-, PR-positive/negative status; HER2+/-, HER2-positive/negative status.

The median CSS for those who received PTR was 39 months (95% CI, 37 months to 41 months) and 22 months (95% CI, 21 months to 24 months) for those who did not (*χ²* = 258, *P* <.001) (**Figure 4B**). Using the matched population, we performed univariable Cox regression for CSS (method analogous to the OS analysis). After multivariable risk adjusting in the Cox regression analysis, PTR was a statistically significant protective factor for CSS (HR, 0.64; 95% CI, 0.59 to 0.67, *P* <.001) (**Table 4**). Besides, tumor grade, age, marital status, tumor size, N stage, ER status, PR status, HER2 status, the receipt of radiotherapy and chemotherapy were validated as independent risk or protective factors as well. We then examined multicollinearity among the candidate variables using variance inflation factors (VIFs); The results are detailed in **Supplementary Table 2**. All VIFs were ≤ 2.5, well below the conventional threshold of 5, indicating only mild collinearity, and no variable was removed on these grounds.

**Table 4:** The results of univariable and multivariable Cox regression for clinical characteristics in cancer-specific survival.

Variable	Univariable			Multivariable		
	Crude HR	95%CI	*P*	Adjusted HR	95%CI	*P*
Age			<.001			
<45	[reference]			[reference]		
45-65	1.231	[1.122,1.349]	<.001	1.155	[1.052,1.269]	.002
>65	1.706	[1.549,1.878]	<.001	1.473	[1.328,1.634]	<.001
Race			.85			
White	[reference]					
Black	1.324	[1.221,1.435]	<.001			
Asian	0.816	[0.726,0.918]	.001			
American India	0.897	[0.636,1.264]	.53			
Marital status			<.001			
Married	[reference]			[reference]		
Single	1.255	[1.160,1.357]	<.001	1.252	[1.157,1.355]	<.001
Divorced/Separated/Widowed	1.416	[1.315,1.525]	<.001	1.205	[1.116,1.301]	<.001
Unknown	1.227	[1.057,1.424]	.007	1.215	[1.045,1.411]	.01
Laterality			.13			
Left	[reference]					
Right	0.948	[0.891,1.009]	.095			
Single-sided but unknown	1.448	[0.778,2.696]	.24			
Paired sides	0.860	[0.686,1.077]	.19			
Histology			.11			
IDC	[reference]					
ILC	1.029	[0.928,1.141]	.59			
Mixed	0.800	[0.707,0.905]	<.001			
Others	1.166	[1.067,1.274]	.001			
Grade			<.001			
1	[reference]			[reference]		
2	1.065	[0.906,1.251]	.45	1.197	[1.036,1.383]	.02
3-4	1.424	[1.216,1.667]	<.001	1.570	[1.357,1.817]	<.001
Unknown	1.171	[0.996,1.377]	.06	1.291	[1.103,1.512]	.001
ER			<.001			
ER+	[reference]			[reference]		
ER-	1.543	[1.445,1.649]	<.001	1.395	[1.256,1.548]	<.001
PR			<.001			
PR+	[reference]			[reference]		
PR-	1.480	[1.391,1.573]	<.001	1.294	[1.190,1.408]	<.001
HER2			<.001			
HER2+	[reference]			[reference]		
HER2-	1.525	[1.373,1.693]	<.001	1.731	[1.550,1.933]	<.001
Unknown	1.752	[1.584,1.938]	<.001	1.806	[1.627,2.004]	<.001
Subtype			<.001			
HR+/HER2-	[reference]			[reference]		
HR+/HER2+	0.706	[0.618,0.806]	<.001	0.735	[0.641,0.844]	<.001
HR-/HER2-	2.180	[1.930,2.463]	<.001	1.366	[1.175,1.587]	<.001
HR-/HER2+	0.820	[0.704,0.957]	.012	0.518	[0.433,0.619]	<.001
Unknown	1.315	[1.224,1.414]	<.001	1.125	[1.039,1.219]	.004
T			<.001			<.001
0	[reference]			[reference]		
1	1.042	[0.841,1.290]	.71	1.505	[1.200,1.866]	<.001
2	1.077	[0.880,1.319]	.47	1.658	[1.335,2.059]	<.001
3	1.229	[0.998,1.512]	.052	1.908	[1.528,2.383]	<.001
4	1.637	[1.346,1.990]	<.001	2.217	[1.799,2.733]	<.001
X	1.522	[1.237,1.872]	<.001	1.685	[1.358,2.090]	<.001
N			<.001			
0	[reference]			[reference]		
1	0.978	[0.896,1.067]	.62	1.011	[0.924,1.106]	.08
2	0.935	[0.836,1.045]	.24	1.026	[0.913,1.152]	.67
3	1.002	[0.908,1.105]	.97	0.990	[0.893,1.097]	.85
X	1.579	[1.420,1.756]	<.001	1.307	[1.170,1.460]	<.001
Nodal Status			<.001			Not included
Unknown	[reference]					
Internal mamary node(s)	1.065	[0.756,1.502]	.72			
Micrometastasized axillary lymph node(s)	0.473	[0.334,0.670]	<.001			
Mobile axillary lymph node(s)	0.862	[0.786,0.946]	.002			
Fixed axillary lymph node(s)	1.068	[0.971,1.175]	.18			
Metastasis			.67			
Only bone metastases	[reference]					
Only brain metastases	2.480	[1.419,4.335]	.001			
Only liver metastases	1.034	[0.683,1.567]	.87			
Only lung metastases	1.058	[0.771,1.454]	.73			
Oligometastases (num<4)	1.116	[0.824,1.509]	.48			
Multplemetastases (num>=4)	1.781	[1.289,2.461]	<.001			
Unknown	1.131	[0.842,1.518]	.41			
Size			<.001			
Unknown	[reference]			[reference]		
<1cm	0.698	[0.578,0.844]	<.001	0.836	[0.691,1.013]	.07
1-2cm	0.735	[0.653,0.828]	<.001	0.907	[0.802,1.025]	.12
2-3cm	0.691	[0.614,0.777]	<.001	0.900	[0.796,1.018]	.09
3-4cm	0.773	[0.685,0.872]	<.001	0.946	[0.835,1.071]	.38
4-5cm	0.842	[0.743,0.956]	.008	1.035	[0.909,1.180]	.60
5-8cm	0.901	[0.817,0.995]	.04	1.093	[0.985,1.213]	.09
>8cm	1.084	[0.990,1.187]	.08	1.261	[1.145,1.389]	<.001
Surgery			<.001			
No	[reference]			[reference]		
Yes	0.609	[0.573,0.648]		0.637	[0.593,0.685]	<.001
Radiotherapy			<.001			
No	[reference]			[reference]		
Yes	0.628	[0.583,0.676]	<.001	0.809	[0.744,0.879]	<.001
Chemotherapy			<.001			
No	[reference]			[reference]		
Yes	0.693	[0.652,0.737]	<.001	0.685	[0.637,0.736]	<.001
Treatment Type			<.001			Not included
No therapy	[reference]					
Surgery only	0.682	[0.611,0.760]	<.001			
Radiation only	0.587	[0.425,0.812]	.001			
Chemotherapy only	0.749	[0.688,0.817]	<.001			
Surgery and chemotherapy	0.514	[0.466,0.568]	<.001			
Surgery and radiation	0.573	[0.495,0.663]	<.001			
Trimodality	0.404	[0.365,0.448]	<.001			
Systemic therapy			<.001			Not included
No systemic therapy	[reference]					
Systemic therapy alone	0.882	[0.284,2.737]	.83			
Surgery after systemic therapy	0.517	[0.463,0.578]	<.001			
Surgery before systemic therapy	0.598	[0.552,0.648]	<.001			
Surgery before and after systemic therapy	0.400	[0.351,0.457]	<.001			
Unknown	0.871	[0.801,0.947]	.001			

HR, hazard ratio.

Data source: Female patients diagnosed with stage IV breast cancer from 2005 to 2015 in the SEER database. ER+/-, ER-positive/negative status; PR+/-, PR-positive/negative status; HER2+/-, HER2-positive/negative status.

Our results indicated that the median OS and median CSS have been prolonged to nearly one and a half years in patients who underwent PTR. After adjusting for these variables in both univariable and multivariable analyses, our study demonstrates that surgery is independently associated with improved survival probability. This outcome supports the conclusion that PTR may be beneficial to the survival prognosis of patients with stage IV BC.

### Subgroup analysis

**Figure 5:**
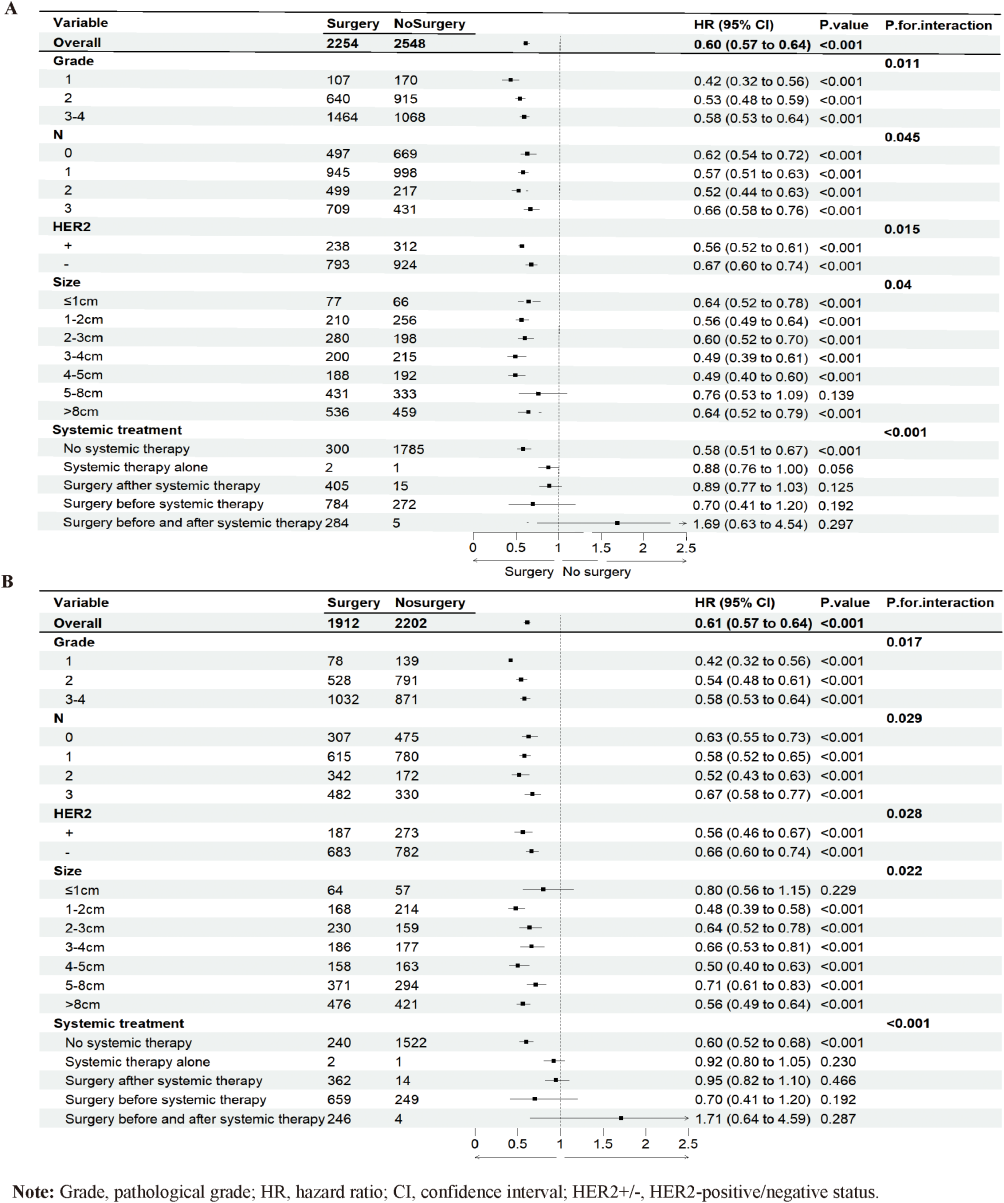
Subgroup analysis of Grade, N stage, HER2 status, tumor size and systemic treatment. **(A)** Based on overall survival. **(B)** Based on cancer-specific survival. Grade, pathological grade; HR, hazard ratio, CI, confidence interval; HER2+/-; HER2-positive/negative status.

The previous data analysis has confirmed that PTR is an independent factor for the survival of patients with stage IV BC. We further conducted subgroup analyses based on patient characteristics to explore their interactions with PTR, with the aim of identifying patient populations in whom the association between PTR and survival outcomes may differ. We first analyzed whether each variable has an interaction with the surgery. Within each subgroup, survival outcomes were compared between patients who underwent surgery and those who did not, in order to assess the strength of the association between PTR and survival across different patient populations. In the subgroup analysis of patient OS, we found that pathological grade, N stage, HER2 status, and tumor size have significant interactions with surgery (*P* for interaction <.05) (**Figure 5A**). Within the subgroup of pathological grade, an association between PTR and improved OS was observed across all pathological grade subgroups, while grade 1 patients have the highest correlation between surgery and OS (HR, 0.42; 95% CI, 0.32 - 0.56). In terms of N stage, PTR and OS prolongation are most correlated in stage N2 patients (HR, 0.52; 95% CI, 0.44 - 0.63). Among HER2 status, we observed that HER2-positive patients (HR, 0.56; 95% CI, 0.52-0.61) demonstrated a stronger association between PTR and improved survival than HER2-negative patients (HR, 0.67; 95% CI, 0.60 - 0.74). Regarding tumor size, when the primary tumor was smaller than 5 cm, surgical resection was associated with improved overall survival. The strongest association was observed among patients with tumors measuring 3–4 cm (HR, 0.49; 95% CI, 0.39 - 0.61) and 4–5 cm (HR, 0.49; 95% CI, 0.40 - 0.60). When the tumor size is in the 5–8 cm range, there is no significant difference in overall survival between patients who did and did not receive PTR (HR, 0.76; 95% CI, 0.53 – 1.09). Similarly, in the CSS subgroup analysis, the strongest association between PTR and improved CSS was observed among patients with N2 disease (HR, 0.52; 95% CI, 0.43–0.63). In addition, HER2-positive patients who underwent PTR demonstrated a stronger association with improved CSS (HR, 0.56; 95% CI, 0.46–0.67) than HER2-negative patients. In terms of tumor size, the association between PTR and improved overall survival was most pronounced in patients with tumors measuring 1–2 cm (HR, 0.48; 95% CI, 0.39–0.58), followed by those with tumors measuring 4–5 cm (HR, 0.50; 95% CI, 0.40–0.63). A significant association between PTR and improved overall survival was also observed in patients with tumors measuring 5–8 cm. Similar to the findings in OS, grade 1 patients exhibit a stronger correlation between surgical intervention and CSS (HR, 0.42; 95% CI, 0.32 - 0.56) (**Figure 5B**).

### Survival outcomes stratified by systemic treatment and treatment type

**Figure 6:**
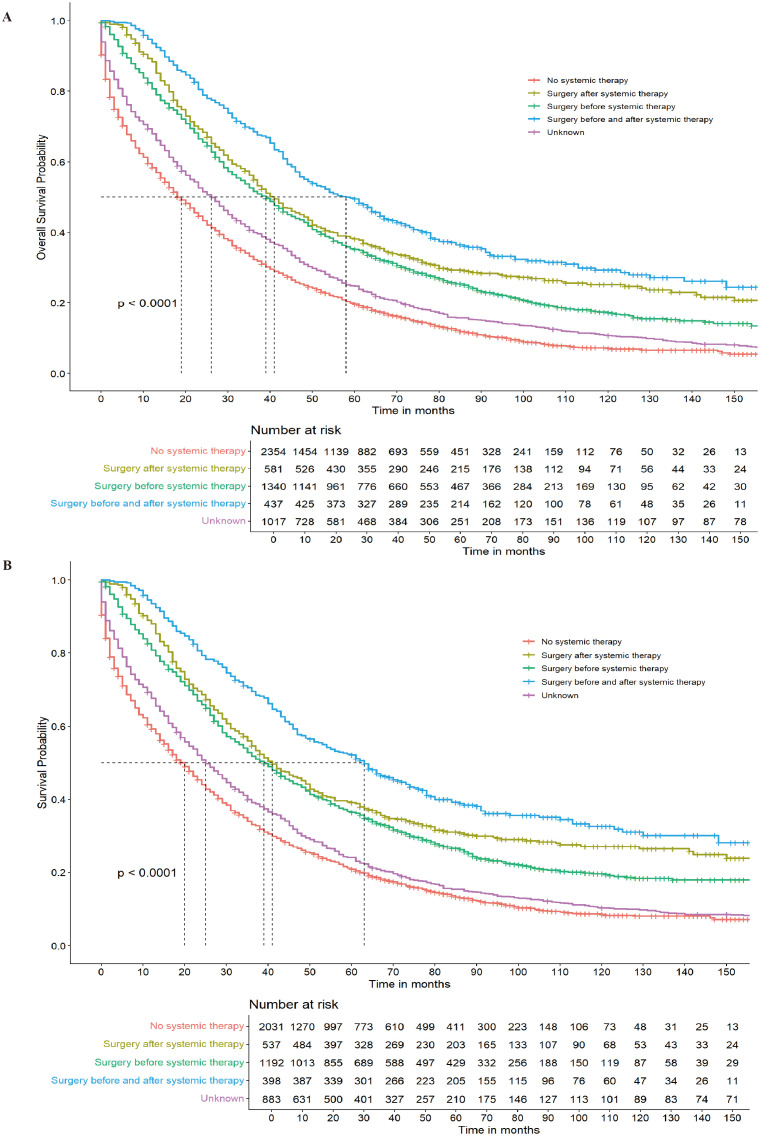
Kaplan-Meier-curves for overall and cancer-specific survival in patients with and without PTR stratified by systemic treatment. **(A)** Overall survival. **(B)** Cancer-specific survival.

Specifically, our subgroup analysis revealed a highly significant interaction between the systemic treatment subgroup and the receipt of PTR (*P* for interaction <.001) (**Figure 5**). Comparing patients who underwent surgery with those who did not, in the subgroup of patients who did not receive systemic treatment, using patients who neither received systemic treatment nor surgery as the control group, the results indicate that patients who underwent surgery were associated with significantly improved overall survival (HR, 0.58; 95% CI, 0.51 - 0.67), and similar results were observed in CSS (HR, 0.60; 95% CI, 0.52 - 0.68). The difference in OS was not significant for BC patients who underwent systemic treatment before, after, or around surgery (*P* >.05). The survival curves of the subgroups treated with systemic therapy showed that the best OS and CSS were observed in patients who received systemic therapy both prior to and following surgery (**Figure 6**). In stark contrast, the poorest survival outcomes were noted in the group that did not undergo systemic treatment, with the results being statistically significant (OS: *χ²* = 424, *P* <.001; CSS: *χ²* = 375, *P* <.001).

**Figure 7:**
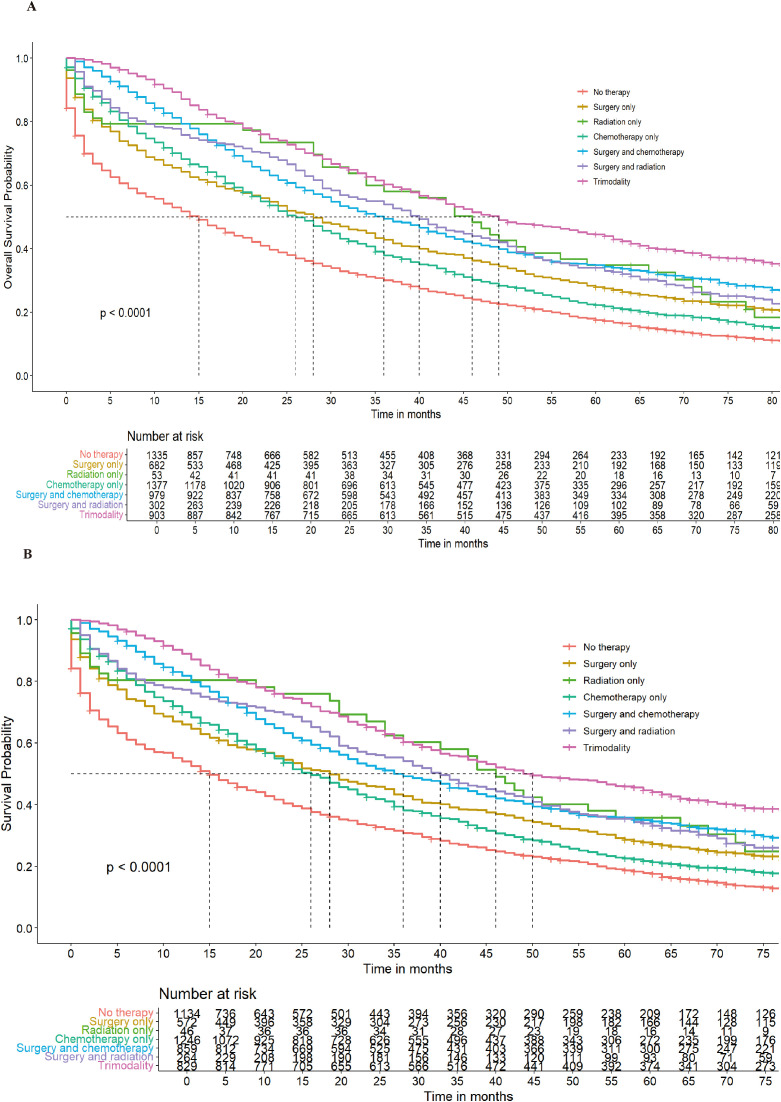
Kaplan-Meier-curves for overall and cancer-specific survival in patients with and without PTR stratified by treatment type. **(A)** Overall survival. **(B)** Cancer-specific survival.

Based on stratifying patients by the treatment type they received, it was revealed that trimodality therapy is associated with the best OS and CSS, with the non-surgery cohort exhibiting the poorest survival (OS: *χ²* = 457, *P* <.001; CSS: *χ²* = 391, *P* <.001) (**Figure 7**).

**Figure 8:**
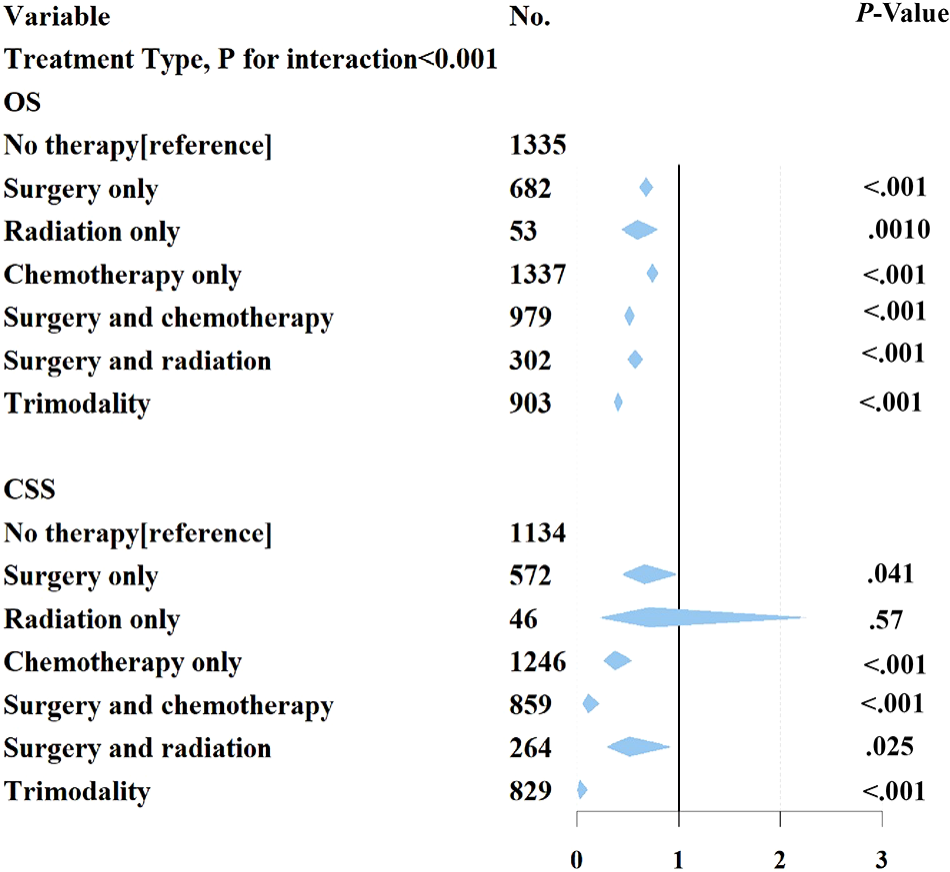
Subgroup analysis of treatment types based on overall survival and cancer-specific survival. The data on the far right of the graph represents the p-value, with the interval indicating the hazard ratio and the 95% confidence level. OS, overall survival; CSS, cancer-specific survival.

Furthermore, subgroup analyses revealed a highly significant interaction between PTR, chemotherapy, and radiotherapy with respect to their associations with survival outcomes. (*P* for interaction <.001) (**Figure 8**). The strongest association with favorable OS was observed in the trimodality therapy group (HR, 0.40; 95% CI, 0.37 - 0.44), followed by the bimodality therapy combining chemotherapy and PTR (HR, 0.51; 95% CI, 0.47 - 0.56), and then the addition of radiation therapy to PTR (HR, 0.57; 95% CI, 0.50 - 0.65). Radiation therapy alone (HR, 0.59; 95% CI, 0.44 - 0.80) had a greater impact on OS compared to PTR alone (HR, 0.55; 95% CI, 0.50 - 0.60), while chemotherapy alone (HR, 0.74; 95% CI, 0.68 - 0.80) had the least impact on OS. Correspondingly, the strongest association with favorable CSS was observed in the trimodality therapy group (HR, 0.03; 95% CI, 0.01 - 0.10), followed by the bimodality therapy combining chemotherapy and PTR (HR, 0.11; 95% CI, 0.06 - 0.22), and then the addition of radiation therapy to PTR (HR, 0.52; 95% CI, 0.29 - 0.92). Chemotherapy alone (HR, 0.37; 95% CI, 0.26 - 0.54) had a more favorable impact on CSS compared to PTR alone (HR, 0.65; 95% CI, 0.45 - 0.98); however, there was no statistically significant difference in CSS for those who received radiation therapy alone.

### Establishing survival prognosis models and web-based application for identifying patients with *de novo* stage IV breast cancer who may be candidates for PTR

**Figure 9:**
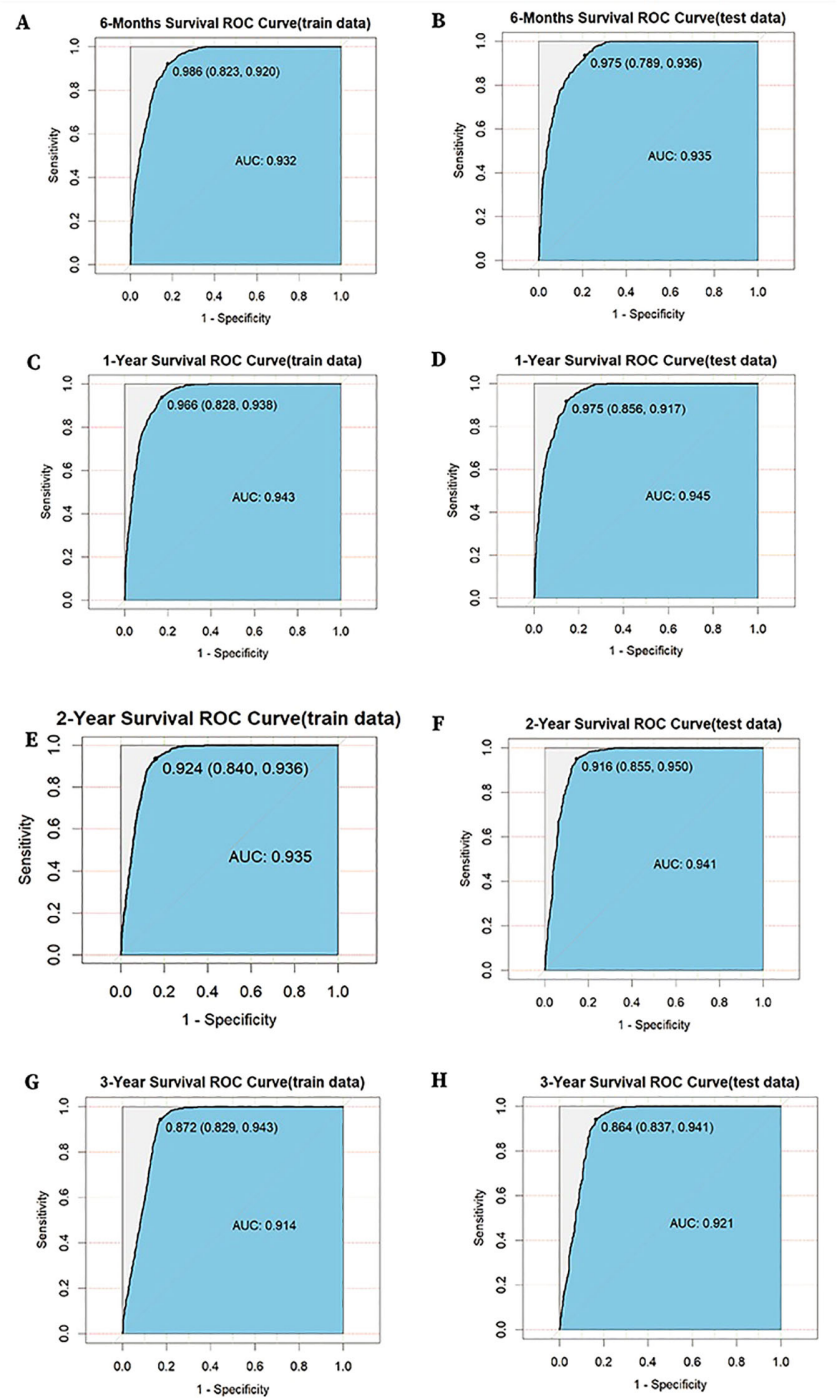
SVM model evaluation. **(A)** ROC curve for the 6-month prognostic model (train data); **(B)** ROC curve for the 6-month prognostic model (test data); **(C)** ROC curve for the 1-year prognostic model (train data); **(D)** ROC curve for the 1-year prognostic model (test data); **(E)** ROC curve for the 2-year prognostic model (train data); **(F)** ROC curve for the 2-year prognostic model (test data); **(G)** ROC curve for the 3-year prognostic model (train data); **(H)** ROC curve for the 3-year prognostic model (test data). ROC, receiver operating characteristic, AUC, arca under the ROC curve; SVM, support vector machine.

In light of the results obtained, we took steps to establish a machine learning prognosis model to predict the OS of the stage IV BC patients. We sorted the patients into training and test data group in a 7:3 ratio. For the training and test sets, we formed the predicted ROC curves and computed the corresponding AUCs. Compared to these machine learning algorithms on test data (**Table 5**), SVM (6-month: AUC = 0.935; 1-year: AUC = 0.945; 2-year:AUC=0.941; 3-year: AUC = 0.921), LR (6-month: AUC = 0.742; 1-year: AUC = 0.724; 2-year: AUC = 0.700; 3-year: AUC = 0.682), RF (6-month: AUC = 0.739; 1-year: AUC = 0.718; 2-year: AUC = 0.702; 3-year: AUC = 0.686), XGBoost (6-month: AUC = 0.747; 1-year: AUC = 0.736; 2-year: AUC = 0.703; 3-year: AUC = 0.664), mlr3 (6-month: AUC = 0.745; 1-year: AUC = 0.736; 2-year: AUC = 0.729; 3-year: AUC = 0.707), SVM model performed exceptionally well in predicting survival of BC patients. The ROC curves and AUC for the SVM model, calculated for the training and test datasets over the periods of six months, one year, two years, and three years, are all presented in **Figure 9**. The calibration curve demonstrated close alignment between predicted probabilities and observed outcomes (**Supplementary Figure 1A**). This was further supported by an overall Brier score of 0.158 (95% CI: 0.144–0.171), indicating acceptable overall accuracy of the probability estimates. Importantly, the model achieved a scaled Brier score of 0.219 (95% CI: 0.168–0.268), which represents a 21.9% relative improvement in predictive accuracy and informative value compared to the null baseline model. Furthermore, DCA demonstrated that the SVM model yielded a higher net benefit across a broad spectrum of reasonable threshold probabilities compared to the default “treat-all” and “treat-none” clinical strategies, indicating that the model could serve as a valuable reference tool for individualized surgical decision-making (**Supplementary Figure 1B**).

**Table 5:** Performance of prognostic models built by 5 machine learning algorithms on training and test data (area under the ROC curve).

Methods	Dataset	Accuracy	6-month of AUC	1-year of AUC	2-year of AUC	3-year of AUC
SVM	Training	0.791	0.932	0.943	0.935	0.941
	Test	0.804	0.935	0.945	0.941	0.921
RF	Training	0.685	0.747	0.747	0.733	0.709
	Test	0.653	0.739	0.718	0.702	0.686
LR	Training	0.686	0.741	0.735	0.717	0.679
	Test	0.693	0.742	0.724	0.700	0.682
mlr3	Training	0.665	0.759	0.751	0.734	0.713
	Test	0.656	0.745	0.736	0.729	0.707
XGBoost	Training	0.670	0.758	0.739	0.713	0.686
	Test	0.657	0.747	0.736	0.703	0.664

ROC, receiver operating characteristic; AUC, area under the ROC curve; SVM, support vector machine; XGBoost, extreme gradient boosting; mlr3, machine learning in R; LR, logistic regression; RF, random forest.

**Figure 10:**
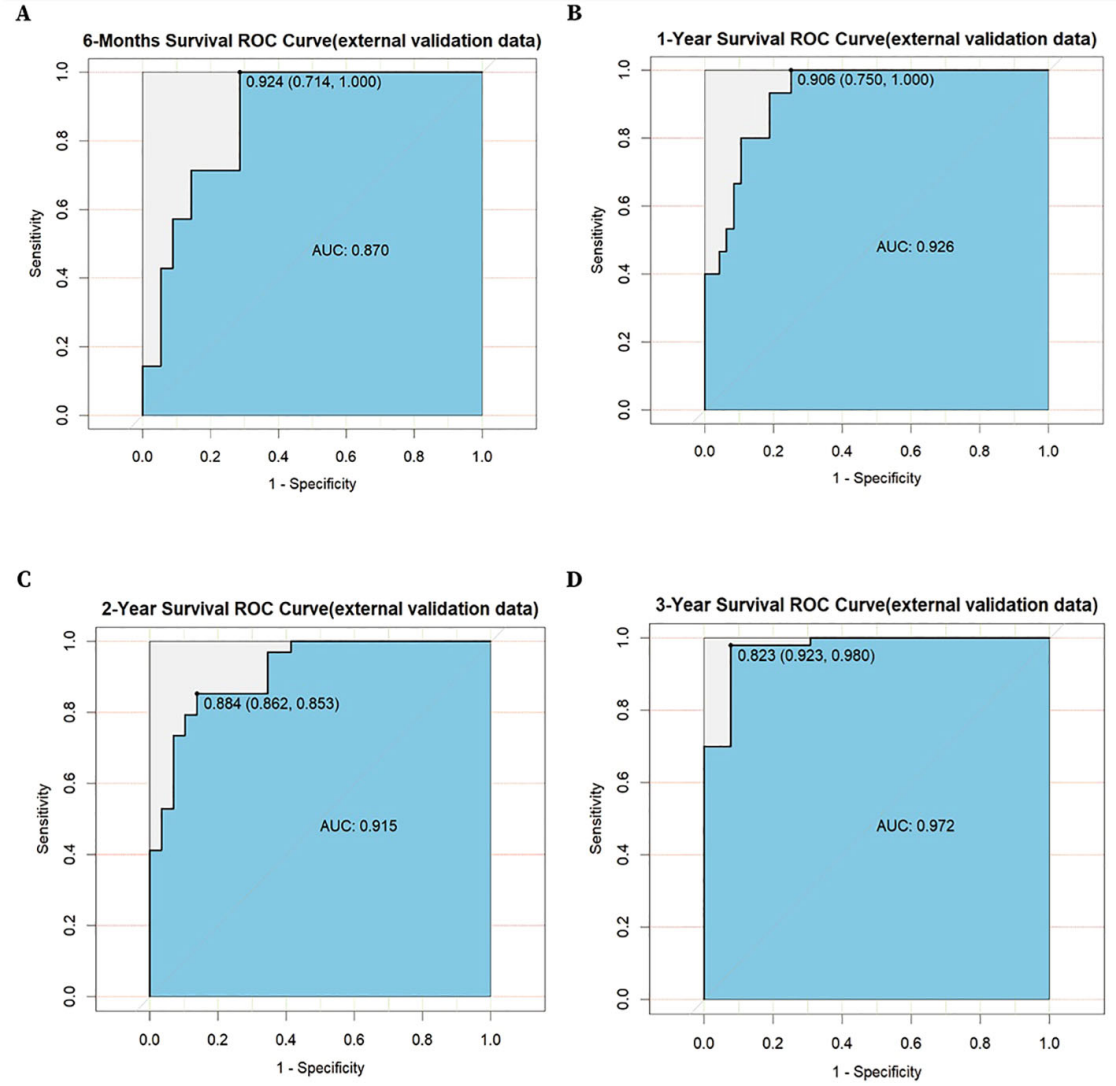
Validation of SVM models from external validation database. **(A)** ROC curve for the 6-months prognostic model; **(B)** ROC curve for the 1-year prognostic model; **(C)** ROC curve for the 2-year prognostic model; **(D)** ROC curve for the 3-year prognostic model. ROC, receiver operating characteristic, AUC, arca under the ROC curve; SVM, support vector machine.

In order to further validate our models, we collected clinical and prognostic information from 67 patients with BCBM from Sun Yet-sen University Cancer Center (**Figure 10**). Although limited by a small external cohort (n=67), the model achieved promising AUCs [6-month: AUC = 0.870,95% bootstrap CI: 0.760-0.957(**Figure 10A**); 1-year: AUC = 0.926, 95% bootstrap CI: 0.860-0.978 (**Figure 10B**); 2-year: AUC = 0.915, 95% bootstrap CI: 0.841-0.971 (**Figure 10C**); 3-year: AUC = 0.972, 95% bootstrap CI: 0.917-0.991 (**Figure 10D**), indicating its potential generalizability pending validation in larger samples.

We performed external validation only; no model updating was conducted. We did not examine heterogeneity in model performance across clusters, as the data were derived from a single cohort.

**Figure 11:**
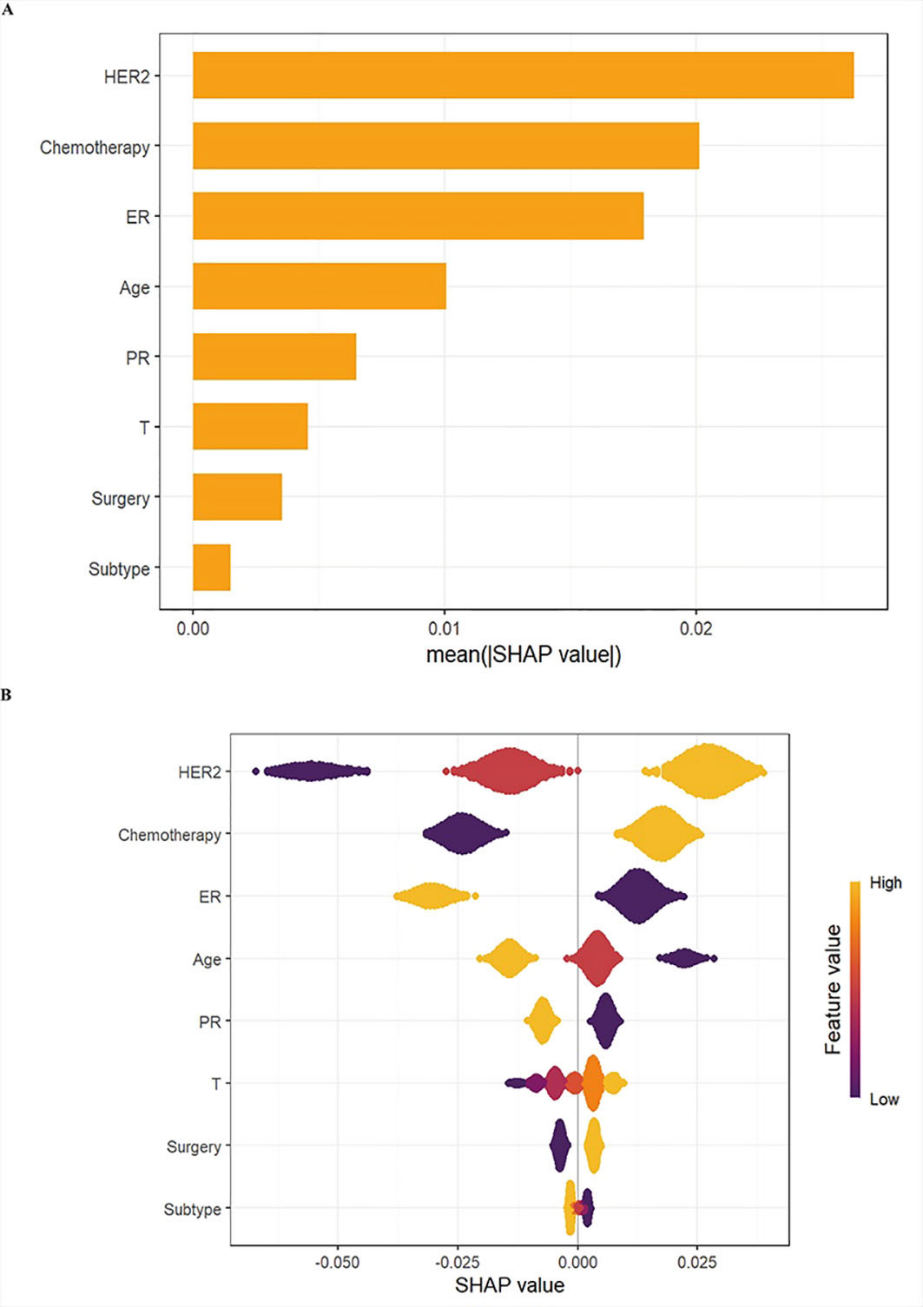
Global model explanation by the SHAP method. **(A)** SHAP summary bar plot. The bar plot illustrates the significance of clinical features affecting survival prognosis; the higher the SHAP value, the greater the association with survival. **(B)** SHAP summary dot plot. A dot is made for SHAP value in the model for each single patient, so each patient has one dot on the line for each feature. The colors of the dots demonstrate the actual values of the features for each patient, as yellow means a higher feature value and purple means a lower feature value.

Additionally, we utilized SHAP to elucidate the outcomes of the final model by calculating the contribution of each variable, facilitating the user’s comprehension of the model. As demonstrated in the SHAP summary plots (**Figure 11**), the overall model’s contribution by each feature is assessed using the average SHAP values, which are displayed in descending order. The top five clinical features impacting the prognosis of patient survival are HER2 status, chemotherapy, ER status, age at diagnosis, and PR status.

### Web-based application development

**Figure 12:**
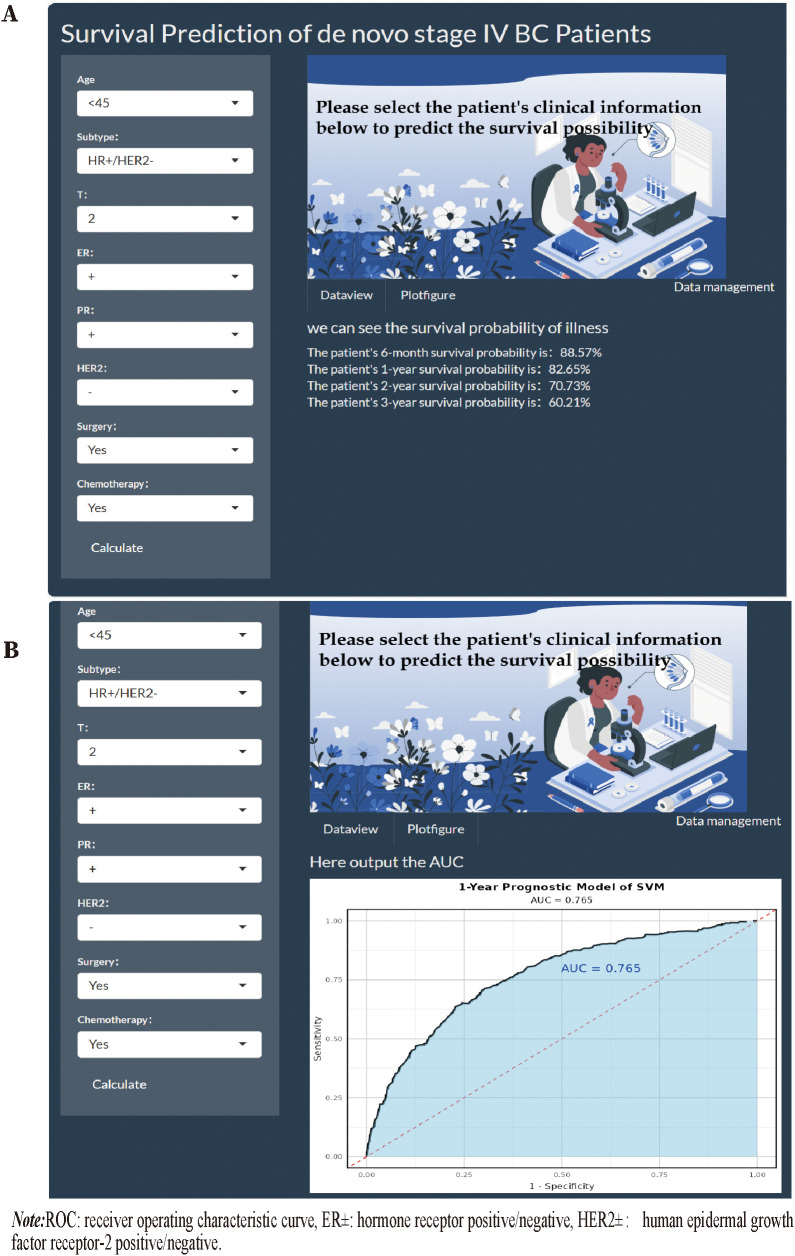
Screenshots of the web application for survival prediction of patients with stage IV breast cancer (https://bcpredictivemodel.shinyapps.io/breast/). **(A)** Presentation of six-month, one-, two-, and three-year survival probability prediction outcomes. **(B)** Display of the ROC curve. ROC, receiver operating characteristic; ER±, hormone receptor positive/negative; HER2±, human epidermal growth factor receptor-2 positive/negative.

To empower researchers and clinicians in leveraging our prognostic models, we have meticulously crafted an intuitive web application utilizing the shiny platform. An online computing platform (https://bcpredictivemodel.shinyapps.io/breast/) for this SVM model is publicly available and free-to-use by doctors and patients. This application features an accessible web interface (as depicted in **Figure 12**), enabling users to effortlessly input the clinical attributes of a new patient sample. Subsequently, the application facilitates the computation of survival probabilities, tailored to the specifics of *de novo* IV stage BC patients. Concurrently, the web tool presents an integrated display of the ROC curve, offering a comprehensive visual assessment of the model’s predictive accuracy.

## Discussion

### Principal results

This study identified clinical characteristics associated with improved survival among stage IV breast cancer patients undergoing primary tumor resection and developed a highly discriminative prognostic model for this population. Our findings indicate that patients with HER2-positive, N2 stage and tumor size less than 5cm are more likely to achieve higher survival probability from surgical treatment. Having identified a suite of predictive risk factors, we developed a ML model capable of forecasting the survival probabilities for patients with *de novo* IV breast cancer. This model demonstrated a remarkable capacity for prognostic prediction, consistently performing well in both internal and external validation processes. Finally, a user-friendly web application was created to facilitate its application in clinical treatment.

As medical science marches forward, the discourse on the advisability of performing PTR for stage IV breast cancer patients is intensifying (9, 27). Some artificial intelligence (AI) assisted surgery decision-making models were developed to assist clinicians in delivering precision medicine (28). Historically, a plethora of retrospective analyses has pointed toward a potential enhancement in the overall survival rates of stage IV breast cancer patients subjected to PTR (9, 28–32). Yet, these retrospective reviews are marred by inherent biases—ranging from age demographics to the timing of surgical intervention, the extent of cancer metastasis, and the criteria for patient selection—which have impeded a granular and precisely accurate synthesis of the data (33). In tandem, a series of prospective studies have delved into the prognostic value of primary tumor resection in the context of stage IV BC. A pivotal Indian study (NCT 0019377), enrolling participants from 2005 to 2012, scrutinized the impact of local surgery on the survival of newly diagnosed stage IV BC patients post systemic therapy (34). The findings suggested that local surgery did not markedly elevate overall survival, challenging the conventional wisdom. Similarly, the ABCSG-28 trial hinted at a trend toward diminished overall survival post-surgery, albeit without statistical significance (13). These prospective endeavors, were also susceptible to methodological flaws, such as skewed patient distribution and a dearth of observational data (33). In our study, we harnessed the SEER public database, spanning from 2005 to 2015, to conduct a meticulously crafted 1:1 propensity score-matched cohort analysis. This approach not only significantly mitigated the confounding effects of bias but also leveraged a more comprehensive set of clinical samples. Our research addresses the pressing demand for up-to-date clinical data, propelling the evolution of clinical treatment strategies in the realm of stage IV breast cancer management.

One of the aims of this study was to identify patient subgroups in which PTR is associated with more favorable survival outcomes. Through univariable, multivariable, and subgroup analyses of the matched cohort, we propose that patients with N2 stage, tumor size less than 5cm, and HER2-positive BC who undergo PTR are more likely to improve their survival probabilities. Our study indicates that grade 1 BC patients who undergo PTR experience improved survival probability. This finding aligns with the general understanding that low-grade tumors, which are characterized by well-differentiated cells that closely resemble normal cells, tend to grow more slowly and have a better prognosis. Studies have shown that low-grade tumors are associated with reduced recurrence and metastasis rates, leading to better overall survival (35, 36).

With the advancement of systemic treatments (endocrine therapy, cytotoxic therapy, anti-HER2 therapy, etc.), the control of metastatic disease has been greatly improved, and the combination of local surgery and systemic treatment may increase the survival rate (28). A study by Lane WO et al. showed that women who underwent surgical treatment after systemic treatment had the greatest survival benefits (9). A multicenter randomized trial named MF07–01 also showed that patients with primary stage IV BC who received locoregional treatment (LRT) had a significant increase in OS at the end of the 10 - year follow – up (12). In our subgroup analysis, we found a very significant interaction between the systemic treatment subgroup and PTR (*P* for interaction <.001). Our results indicate that, among patients who did not receive systemic treatment, an association between surgical treatment and improved survival was observed. (OS: HR, 0.58; CSS: HR,0.60). This suggests that in clinical practice, surgery may be beneficial for the survival of advanced breast cancer patients who refuse systemic therapy. Regrettably, the correlation between PTR and overall survival in stage IV breast cancer patients was not found to be statistically significant. This was true regardless of whether the patients received PTR before surgery, postoperative therapy after surgery, or a combination of both. In addition, our analysis shows that chemotherapy and radiotherapy are both in dependent factors related to the survival of stage IV BC patients. Previous studies have suggested that for patients with locally advanced breast cancer, an integrated treatment plan combining chemotherapy, radiotherapy, and surgery is more conducive to better patient prognosis (37, 38).

Although trimodality therapy produced the most favorable outcomes, patients who received only bimodal treatment (surgical resection plus chemotherapy, without radiotherapy) demonstrated comparatively less pronounced survival benefits. We attribute this observation to several interconnected factors. First, patients with more advanced disease burden, poorer performance status, or unfavorable prognostic characteristics may have been more likely to receive surgery and chemotherapy while remaining unsuitable candidates for additional radiotherapy. Such treatment selection bias may have contributed to worse outcomes in this subgroup. Second, the combination of surgery and chemotherapy may impose substantial treatment-related toxicity, particularly in patients with compromised health status, potentially offsetting part of the therapeutic benefit.

Notably, trimodality therapy was associated with the most favorable survival outcomes in our analysis. However, these findings should be interpreted with caution. Patients classified into the trimodality group were required to survive long enough to receive surgery, chemotherapy, and radiotherapy, creating the potential for immortal time bias. Because the SEER database does not provide precise treatment dates, we were unable to model treatment exposure as a time-varying covariate using a time-dependent Cox regression approach. Consequently, part of the apparent survival advantage observed in the trimodality group may reflect guaranteed survival time before completion of all treatment modalities rather than the full therapeutic effect itself. Therefore, the exceptionally low hazard ratios observed for trimodality therapy should be interpreted as demonstrating a strong association with improved survival rather than definitive evidence of a causal treatment effect. Nevertheless, even after accounting for this limitation, the consistently superior outcomes observed among patients receiving comprehensive multimodality treatment may suggest that appropriately selected patients derive meaningful benefit from combined local and systemic therapeutic strategies. Further prospective studies with detailed treatment timelines and time-dependent analyses are warranted to clarify the true magnitude of benefit associated with trimodality therapy and to optimize treatment selection for patients with *de novo* stage IV breast cancer.

The other purpose of this study is to develop a clinical prediction model for stage IV BC patients. For stage IV BC patients, the battle against the disease is not about cure but about maximizing the duration of survival—a quest for extending life’s narrative. However, there is still a lack of reliable survival prediction models in current clinical practice. In comparison to traditional statistical approaches, machine learning offers a sophisticated alternative for the analysis of clinical datasets, enabling the construction of robust risk models and the reclassification of patient groups (39, 40). The application of machine learning in the prognostic prediction of breast cancer patients has been extensively explored in recent studies (41–46), with a variety of algorithms being employed, including LR, XGBoost, RF, K-Nearest Neighbors (KNN), SVM and Bayesian classifiers, etc. Although there are many studies that have developed survival prediction models (41, 47–49), there are some problems: (1) the accuracy is not high (<0.7); (2) the deployment of machine learning algorithms that do not align well with the nuances of clinical survival data. After identifying the independent factors related to patient survival, we developed a more accurate SVM model with 8 features to address the above issues. Among 5 ML models, the SVM model had the highest accuracy and the best AUC value. SVMs are a more recent approach of ML methods applied in the field of cancer prediction/prognosis, which can map the input vector into high dimensional feature spaces, separate the input data into reliable classifications (20, 50). The performance variation between models observed in our study highlights that algorithm efficacy is highly dependent on dataset characteristics, a principle observed in other computational biology contexts (51). Our feature importance analysis (**Supplementary Table 4**) demonstrates that each model leveraged the predictor space in fundamentally different ways, which likely contributed to their divergent generalization outcomes. Notwithstanding these algorithmic differences, our final model demonstrated good performance in both internal and external validation. Representative failure cases are detailed in **Supplementary Table 3** to provide a comprehensive view of its capabilities.

Furthermore, we employed the SHAP to elucidate the model’s inner workings through a global explanation, painting a comprehensive picture of the model’s overall functionality. The impetus behind crafting a survival prediction model is to enhance its clinical utility. Yet, many newly introduced models face the drawback of being difficult to disseminate and apply (52). Leveraging the power of the Shiny programming framework, we have developed a web platform for this predictive model, facilitating its global adoption and application in a more accessible and user-friendly manner.

The advent of digital health solutions has witnessed a surge in web-based applications for disease and survival prediction, leveraging the power of machine learning algorithms to analyze complex medical data (50, 53, 54). Our study contributes to this field by developing an interactive web application using R’s Shiny package. This application provides a platform to access our prognostic model, which estimates survival probabilities for stage IV breast cancer patients based on a set of clinical features, including surgery status. The significance of our study extends beyond the technical implementation. This tool offers data-informed reference information that can contribute to the prognostic assessment and clinical evaluation of potential surgical candidates. By providing estimated survival probabilities based on retrospective patterns, our web application can inform discussions between healthcare providers and patients regarding overall prognosis and management strategies. Furthermore, the application’s ability to delineate prognostic patterns can also be a valuable asset for research, enabling investigators to explore the dynamics of disease progression in relation to patient management pathways. Future work includes prospective validation of this tool in multi-center settings and refinement of the algorithm with the integration of additional molecular biomarkers.

### Limitations

There are several limitations to this study. First, despite the use of PSM, residual confounding may persist because of the retrospective design and unmeasured factors. Comparisons involving multimodal treatments are also susceptible to immortal time bias and confounding by indication. Patients receiving trimodality therapy may have had more favorable baseline characteristics and better treatment tolerance, potentially leading to an overestimation of the observed survival advantage. Moreover, the lack of detailed treatment timeline data in SEER precluded time-dependent analyses.

Second, the SEER database does not provide information on the specific agents, timing, or duration of systemic therapies. In particular, data regarding anti-HER2 treatment were unavailable, preventing us from distinguishing the independent association of PTR from that of HER2-targeted therapy among HER2-positive patients. Furthermore, quality-of-life measures, symptom burden, and local disease control outcomes were unavailable; therefore, our model and web application are limited to survival prediction.

Third, although the landmark-based binary classification framework enabled clinically intuitive prediction of fixed-time survival probabilities, survival-specific machine learning methods that explicitly account for censoring were not evaluated. Future studies should compare these approaches to determine whether additional predictive gains can be achieved while making more efficient use of censored observations.

Finally, the external validation cohort was small (n=67), limiting the statistical power and stability of this assessment despite its value for generalizability. Therefore, claims regarding the model’s robustness and generalizability are primarily supported by internal validation, and the external results should be seen as an encouraging but non-definitive indicator until validated in larger, multi-center populations.

## Conclusion

In conclusion, this study demonstrates that PTR significantly improves survival outcomes for stage IV breast cancer patients, especially those with tumors ≤5 cm, N2 status, and HER2 overexpression. Our machine learning model, leveraging eight clinical indicators, accurately predicts survival probabilities for these patients. It has shown high accuracy in internal validation and promising consistency in a preliminary external cohort, demonstrating its capability to identify potential candidates for surgical intervention. We anticipate future prospective clinical studies to further validate its efficacy and explore its broader clinical benefits.

## Data Availability

The data that support the findings of this study were obtained from the Surveillance, Epidemiology, and End Results (SEER) Program public-use database. These data are available upon request to the corresponding authors, who will facilitate access in accordance with SEER data use agreements. For the external validation set of clinical data, due to ethical restrictions and to protect patient confidentiality, the data are not publicly shared. Requests to access the datasets should be directed to 202130520285@mail.scut.edu.cn.

## Supplementary material

Supplementary Figure 1 Assessment of model calibration performance and clinical utility. **(A)** Overall calibration curve of the prognostic model. **(B)** Decision curve analysis (DCA) of the prognostic model.

Supplementary Table 1 Sensitivity analysis comparing baseline characteristics of included versus excluded cohorts.

Supplementary Table 2 Variance Inflation Factor (VIF) analysis for assessing multicollinearity.

Supplementary Table 3 Detailed descriptions of representative model failure cases.

Supplementary Table 4 Comparative analysis of feature importance across machine learning models.
