# Supplementary figures and images for: Development and validation of a prognostic model for stage IV breast cancer based on primary tumor resection with machine learning methods: retrospective cohort study
Source: Front Endocrinol (Lausanne). 2026 Jul 13;17:1871537. doi: 10.3389/fendo.2026.1871537 (PMC13402208; doi:10.3389/fendo.2026.1871537)

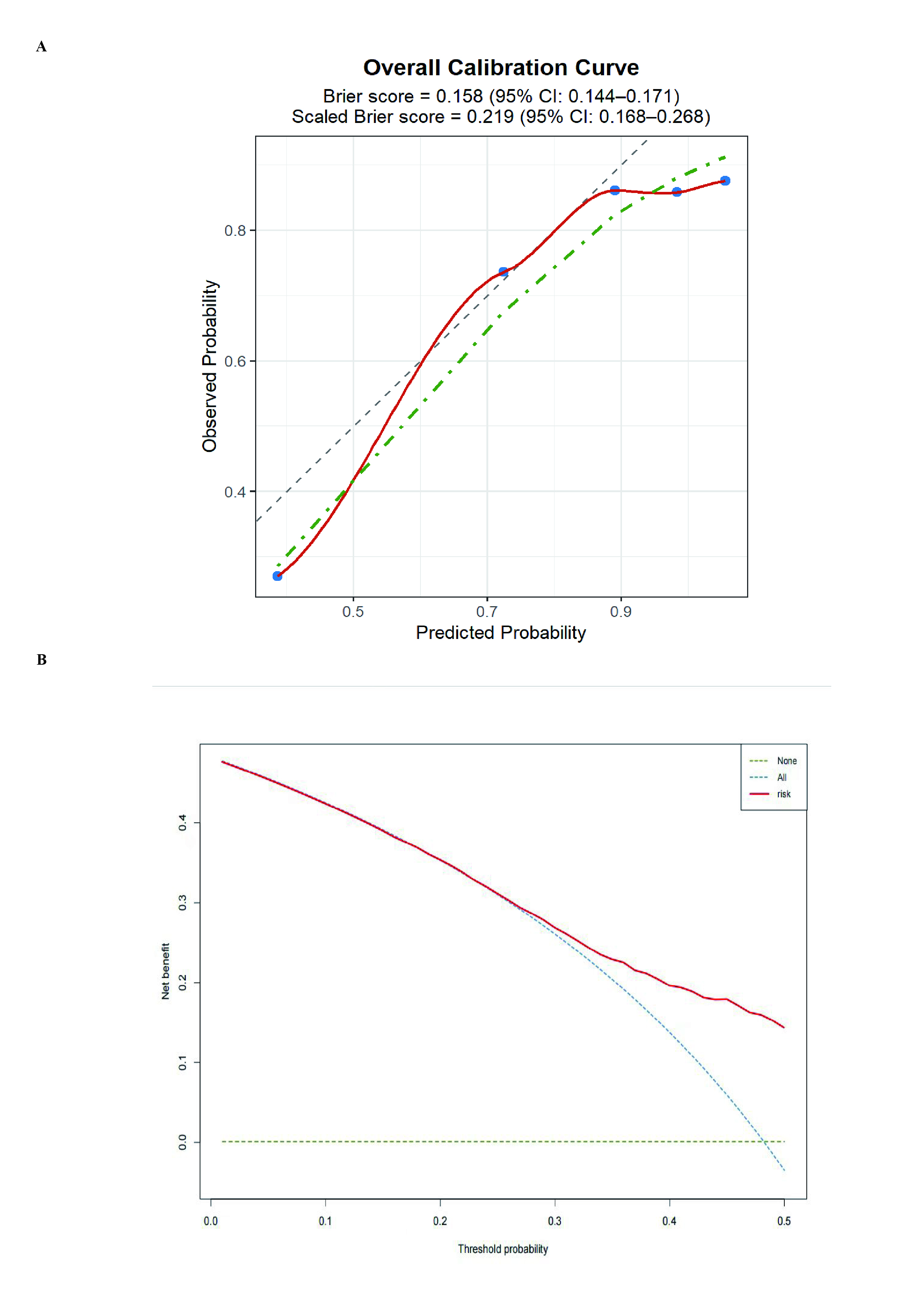

Supplement: Supplementary Figure 1 — Assessment of model calibration performance and clinical utility. (A) Overall calibration curve of the prognostic model. (B) Decision curve analysis (DCA) of the prognostic model. [file Image1.jpg]
